# Large Scale Metabolic Profiling identifies Novel Steroids linked to Rheumatoid Arthritis

**DOI:** 10.1038/s41598-017-05439-1

**Published:** 2017-08-22

**Authors:** Noha A. Yousri, Karim Bayoumy, Wessam Gad Elhaq, Robert P. Mohney, Samar Al Emadi, Mohammed Hammoudeh, Hussein Halabi, Basel Masri, Humeira Badsha, Imad Uthman, Robert Plenge, Richa Saxena, Karsten Suhre, Thurayya Arayssi

**Affiliations:** 1 Physiology and Biophysics, Weill Cornell Medical College - Qatar, Education City, Doha, Qatar; 2 Computer and Systems Engineering, Faculty of Engineering, Alexandria University, Alexandria, Egypt; 3 Research Division, Weill Cornell Medical College-Qatar, Education City, Doha, Qatar; 4 Department of Internal Medicine, Hamad Medical Corporation, Doha, Qatar; 5 Metabolon Inc., Durham, NC USA; 6 Division of Rheumatology, Department of Medicine, Hamad Medical Corporation, Doha, Qatar; 7 Department of Internal Medicine, Division of Rheumatology, King Faisal Specialist Hospital & Research center, Jeddah, Kingdom of Saudi Arabia; 8 Jordan Hospital, Amman, Jordan; 9 Dr. Humeira Badsha Medical Center, Dubai, UAE; 10 American University of Beirut, Faculty of Medicine, Beirut, Lebanon; 11 Immunology & Inflammation, Celgene, Cambridge, MA USA; 12 Center for Genomic Medicine, Department of Anesthesia, Critical Care and Pain Medicine Massachusetts General Hospital, Harvard Medical School, Boston, MA USA; 13 Program in Medical and Population Genetics, Broad Institute, Cambridge, MA USA; 14 Department of Medicine, Weill Cornell Medical College-Qatar, Education City, Doha, Qatar

## Abstract

Recent metabolomics studies of Rheumatoid Arthritis (RA) reported few metabolites that were associated with the disease, either due to small cohort sizes or limited coverage of metabolic pathways. Our objective is to identify metabolites associated with RA and its cofounders using a new untargeted metabolomics platform. Moreover, to investigate the pathomechanism of RA by identifying correlations between RA-associated metabolites. 132 RA patients and 104 controls were analyzed for 927 metabolites. Metabolites were tested for association with RA using linear regression. OPLS-DA was used to discriminate RA patients from controls. Gaussian Graphical Models (GGMs) were used to identify correlated metabolites. 32 metabolites are identified as significantly (Bonferroni) associated with RA, including the previously reported metabolites as DHEAS, cortisol and androstenedione and extending that to a larger set of metabolites in the steroid pathway. RA classification using metabolic profiles shows a sensitivity of 91% and specificity of 88%. Steroid levels show variation among the RA patients according to the corticosteroid treatment; lowest in those taking the treatment at the time of the study, higher in those who never took the treatment, and highest in those who took it in the past. Finally, the GGM reflects metabolite relations from the steroidogenesis pathway.

## Introduction

Rheumatoid Arthritis is a chronic inflammatory joint disease characterized by synovial proliferation that results in joint destruction. Early diagnosis and treatment are important to decrease complications but remain a difficult target to achieve^1^. The use of antibodies against cyclic citrullinated peptides and/or proteins (ACPA) can be useful for the diagnosis of RA, but not all patients with RA are seropositive for ACPA, and the antibody may not be present early in the disease. Lately, profiling of metabolites has been shown to be useful in detecting altered metabolism in several diseases, such as diabetes^2–4^, obesity^5–7^, and osteoarthritis^8^. In RA in particular, metabolomics was helpful in providing new insights into the pathogenesis of the disease, discriminating between RA and other musculoskeletal disorders, classifying patients according to disease activity and predicting response to treatment^1, 9–11^. The importance of metabolic profiling is not limited to finding biomarkers that can be used for diagnosis. It extends to finding groups of metabolites that interact with each other, which facilitates finding biological pathways that help understanding the disease pathogenesis. Most studies to date involve investigating a small number of metabolites in a rather small cohort^1, 9, 10, 12^.

This study investigates the metabolic profiles of RA patients compared to healthy controls, all of an Arab ancestry using a set of ~1000 metabolites, obtained by an untargeted metabolomics profiling technology. First, we identify metabolites associated with RA compared to controls, and second, using only the RA patient cohort, we investigate the association of the identified RA metabolites with medication and other cofounders present only for RA patients. We also investigate the accuracy of classifying RA patients using their metabolic profiles, and the correlations between RA associated metabolites using Gaussian Graphical Models.

## Materials and Methods

A total of 132 RA patients and 104 controls were included in this study. Patients and controls were enrolled from two centers in the Kingdom of Saudi Arabia^13^ and Qatar. Plasma samples were collected from subjects enrolled from KSA and whole blood samples were collected from the subjects enrolled from the center in Qatar. To be eligible for the study, patients had to be of Arab ancestry by self report, have been diagnosed as RA according to 1987 ACR criteria^14^, be ≥18 years of age and able to sign an informed consent. Controls had to be free of autoimmune diseases. Ancestry was classified as self-reported Gulf, Levant or African Arab. In addition to age and self-reported ancestry, we collected information on gender, disease duration, smoking status (both cigarettes and shisha), the presence or absence of rheumatoid factor (RF) and anti-cyclic citrullinated antibodies (Anti-CCP), medications used to treat RA, family history of RA, presence of other autoimmune diseases, diabetes, and vascular comorbidities (Table 1). All samples were stored at −80 °C until analyzed. All subjects had to sign an informed consent. The study and experimental protocols were approved in Saudi Arabia by the King Faisal Specialist Hospital & Research Centre Institutional Review Board Committee – Jeddah, and in Qatar by Hamad Medical Corporation (HMC) Institutional Review Board Committee, and Weill Cornell Medicine-Qatar (WCM-Q) Institutional Review Board Committee, and the joint Institutional Review Board Committee of HMC and WCM-Q. All experiments were performed in accordance with relevant guidelines and regulations.

**Table 1:** Subject Characteristics.

Characteristics	RA (N = 132)	Controls (N = 104)
N	132	104
Age (yrs) (mean + SD)	49.62 +/− 12 yrs	43.19 +/− 13.2 yrs
#Female/total (%)	114/132 (86%)	85/104 (81%)
Duration of RA (yrs) (mean +/− SD)	10.6 yrs +/− 8.6 yrs	N/A
#Smokers (%)	24 (18.2%)	27 (25.9%)
#Seropositive (%)	109/132 (82.6%)	N/A
#Family History of RA	42 (31.8%)	N/A
#Biologics*	56 (43.4%)	N/A
#Corticosteroids	114 (86.6%)	N/A
#with Diabetes (%)	27 (20%)	13 (12.5%)
#with Autoimmune (%) diseases	13 (9.8%)	N/A
#with Vascular comorbidities	15 (11.3%)	4

*Biologics include anti-TNF therapy or Rituximab. N/A indicates not applicable. Smokers indicate current smokers or ever smoked.

### Merged Cohort

Metabolic profiles of two sample batches (a batch is defined according to the center of enrollment), 99 samples from KSA and 137 samples from Qatar, were merged after preprocessing (see below) forming a cohort of 236 subjects (132 cases, 104 controls), and 927 metabolites. The batch effect was used as a covariate in the regression analysis.

### Preprocessing

Outlier values for each metabolite were removed if they fall above 3 standard deviations of the mean value. Metabolites that have less than 50% missing values were then selected for the analysis, resulting in a list of 927 metabolites. Metabolite concentrations are then log-transformed and z-scored.

### Regression Analysis

#### I-RA vs ﻿﻿control

﻿﻿Metabolites were regressed against RA vs controls, using a linear regression model (lm function in R statistical package) including the following covariates: age, gender, diabetes, smoking (including shisha smoking), batch effect, as well as interactions of RA with both gender and batch effect. Details on the algorithm used to incorporate the interactions is given in Supplemental Data.

#### II- RA ﻿patients

For metabolites found associated with RA, we performed feature selection (*step* function in R) only using RA patients’ samples to find factors affecting the metabolite levels. Those factors (features) include the previously indicated factors (age, gender, diabetes, smoking, and batch effect), and adds to them the following factors specific to RA patients (as in Table 1):

1. Presence of antibodies (seropositive and seronegative cases) - Binary trait: Seropositive status was defined by the presence of either positive rheumatoid factor (RF) or positive Anti-CCP (n = 109). Seronegative status was defined by the absence of both RF and Anti-CCP (n = 23 cases).
2. Duration of RA – Numerical trait: mean is 10.6 years and standard deviation is 8.6 years.
3. Family history of RA – Binary trait: 42 patients have family history.
4. Corticosteroid treatment – Numerical trait: Subjects were sorted based on the observation of steroid levels: Level ‘2’ are those who were on corticosteroids at the time of the study (73 RA subjects), level ‘1’ are those who never took corticosteroids (18 RA subjects), and level ‘0’ includes past corticosteroid-treated patients (41 RA subjects).
5. Biologics treatment (include anti-TNF therapy and Rituximab) – Binary trait: based on taking any type of biologics medication in the past or at the time of the study (56 RA patients).
6. Autoimmune disease - Binary trait: 13 patients.
7. Coronary/stroke/vascular disease – Binary trait: 15 patients.

After finding the factors (features) affecting each of the selected metabolites, metabolites were then regressed against those factors only and considering RA ﻿patients only(Supplemental Table 4).

### Pathway enrichment analysis

For finding enrichment of a particular pathway in the obtained list of metabolites associated with RA, we use a fisher exact test in R statistical package considering the four counts: (1) the number of metabolites from a particular pathway in the identified RA metabolites and (2) the number of metabolites from that pathway in  the whole metabolite set, (3) the number of metabolites in other pathways in the set of identified RA metabolites, and (4) the number of metabolites in other pathways in the whole metabolite set.

### Classification Analysis

Orthogonal Partial Least Squares – Discriminant Analysis (OPLS-DA) in SIMCA 14 was used to predict RA samples. Two experiments were conducted. In the first experiment, all 927 metabolites and the covariates (age, gender, batch, smoking, shisha smoking, diabetes) and 236 samples were used to model the prediction. In the second experiment we wanted to investigate which metabolites and covariates can enhance the prediction using OPLS-DA. Therefore, feature selection was done using the step regression function in R (*step* and *glm* functions in R) on the 376 metabolites which pass the threshold of p < 0.02 from the regression model (see above) (the cutoff was based on experimental analysis that showed that higher p-values didn’t achieve better feature sets). Briefly, given the list of metabolites and covariates, a stepwise regression (*step* function in R) performs forward and backward selection of variables, adding and dropping them as to increase the Akaike Information Criterion (AIC), and a logistic regression of case/control (*glm* function in R), against the 376 metabolites and six covariates, is used as the base of that step function. The metabolites and covariates identified from this feature selection were used as input for OPLS-DA. OPLS-DA uses a cross validation technique, where the data is divided into a training and test set multiple times, where the training set is used to learn the model and the test set evaluates the model’s accuracy. This is done multiple times so that the test set spans the whole data set. Results of classification using OPLS-DA are indicated with an accompanying Fisher’s probability value, which is the probability of occurring by chance for the classification results obtained. Metabolites were ranked based on their correlation to the OPLS-DA component (p(corr)), and these ranks were compared to their ranks inferred from the p values of regression analysis (comparing RA to controls), where comparison was done using Fisher’s exact test.

### Gaussian Graphical Modeling (GGM)

Pairwise partial correlations between all 927 metabolites were computed using GeneNET library in R statistical package. Bonferroni significant partial correlations (p <= 0.05/(927 * 926/2)) were used to build the GGM network, where each node in the network presents a metabolite and each edge between two nodes is a significant partial correlation. We further pruned the network by removing metabolites that do not associate with RA (keeping only those that associate with RA at p <= 0.05), and removing edges that connect them to other metabolites. We then selected the largest 4 sub-networks of 11 sub-networks for our analysis.

## Results

### 32 metabolites are identified as associated with RA

A total of 927 metabolic measures were regressed against 132 RA cases and 104 controls. 32 metabolites were found to be Bonferroni significantly (p < 0.05/927 = 5.39 × 10^−5^) associated with RA cases (Table 2, and Supplemental Table 1), and 190 metabolites were found to be associated with RA at a nominal significance (p < 0.05). Among the 32 Bonferroni significant metabolites, 16 metabolites are steroids, with an enrichment p-value of 7.8 × 10^−14^ in the steroid pathway (the most significant ones are: dehydroisoandrosterone sulfate (DHEA-S), 4-androsten-3beta,17beta-diol monosulfate (1) (tentatively renamed by Metabolon as androstenediol (3beta,17beta) monosulfate (1)), 4-androsten-3alpha,17alpha-diol monosulfate (3) (tentatively renamed by Metabolon as androstenediol (3alpha,17alpha) monosulfate (3)), 4-androsten-3beta,17beta-diol disulfate (2) (or androstenediol (3beta,17beta) disulfate (2)), and pregn steroid monosulfate), 11 are unknown (unidentified) metabolites (8 of them are tentatively identified by Metabolon as steroids –see Table 2), 2 metabolites are in the BCAAs pathway (4-methyl-2-oxopentanoate and 3-methyl-2-oxovalerate), and 3 other metabolites, specifically a xenobiotic (iminodiacetate), a dipeptide (prolylglycine), and a glycine-serine-threonine metabolite (sarcosine). Among the 190 metabolites with nominal significance (p < 0.05) are 24 sterols/steroids, 13 in the fatty acid metabolism (long-chain, medium-chain and other fatty acid metabolism), 8 in the leucine, isoleucine and valine pathway, 6 in the phenylalanine and tyrosine pathway, 7 in the purine metabolism (among which inosine and hypoxanthine are hypoxanthine containing) (see Supplemental Table 1).

**Table 2:** 32 Metabolites with Bonferroni significant association to RA compared to controls. Metabolites are sorted on their significance of association to RA.

Metabolite	Subpathway	Superpathway	p value RA	Cases vs Controls	Corticosteroid – p value	OPLS-DA Rank^a^	OPLS-DA Rank^b^
dehydroisoandrosterone sulfate (DHEA-S)	Steroid	Lipid	4.08 × 10^−9^	Down	5.56 × 10^−7^	1	1
X – 11444 (urocortisol glucuronide or cortolone glucuronide)^c^	NA	NA	1.02 × 10^−8^	Down	6.57 × 10^−9^	36	2
X – 12844 (tetrahydrocortisone glucuronide)^c^	NA	NA	1.47 × 10^−8^	Down	6.85 × 10^−9^	27	NA
4-androsten-3beta,17beta-diol monosulfate (1) (androstenediol (3beta,17beta) monosulfate (1))^c^	Steroid	Lipid	1.60 × 10^−8^	Down	5.77 × 10^−6^	5	NA
iminodiacetate (IDA)	Chemical	Xenobiotics	3.40 × 10^−8^	Down	NA	135	25
X – 21364	NA	NA	6.66 × 10^−8^	Down	1.14 × 10^−7^	13	NA
4-androsten-3alpha,17alpha-diol monosulfate (3) (androstenediol (3alpha,17alpha) monosulfate (3))^c^	Steroid	Lipid	9.28 × 10^−8^	Down	6.09 × 10^−5^	6	NA
4-androsten-3beta,17beta-diol disulfate (2) (androstenediol (3beta, 17beta) disulfate (2))^c^	Steroid	Lipid	1.07 × 10^−7^	Down	8.42 × 10^−7^	8	NA
pregn steroid monosulfate*	Steroid	Lipid	2.40 × 10^−7^	Down	6.85 × 10^−8^	3	NA
X – 12846 (11 beta-hydroxyandrosterone glucuronide or 11 beta-hydroxyetiocholanolone glucuronide)^c^	NA	NA	2.61 × 10^−7^	Down	1.24 × 10^−7^	66	NA
X – 11440 (hydroxypregnen-diol disulfate (or pregnenolone-diol disulfate))^c^	NA	NA	2.90 × 10^−7^	Down	7.99 × 10^−7^	14	NA
epiandrosterone sulfate	Steroid	Lipid	4.74 × 10^−7^	Down	2.41 × 10^−6^	4	NA
androsterone sulfate	Steroid	Lipid	5.25 × 10^−7^	Down	2.84 × 10^−5^	2	NA
4-androsten-3beta,17beta-diol disulfate (1)	Steroid	Lipid	1.37 × 10^−6^	Down	1.60 × 10^−4^	12	NA
X – 21470 (11 beta-hydroxyandrosterone disulfate or 11 beta-hydroxyetiocholanolone disulfate)^c^	NA	NA	1.46 × 10^−6^	Down	1.60 × 10^−5^	21	5
X – 21410 (11 beta-hydroxyandrosterone sulfate or 11 beta-hydroxyetiocholanolone sulfate)^c^	NA	NA	2.03 × 10^−6^	Down	3.99 × 10^−4^	20	NA
sarcosine (N-Methylglycine)	Glycine, Serine and Threonine Metabolism	Amino Acid	2.13 × 10^−6^	Down	NA	441	NA
X – 18779	NA	NA	3.19 × 10^−6^	Down	1.28 × 10^−1^	242	26
4-methyl-2-oxopentanoate	Leucine, Isoleucine and Valine Metabolism	Amino Acid	4.82 × 10^−6^	Down	1.52 × 10^−2^	78	NA
X – 17359 (urocortisol glucuronide or cortolone glucuronide)^c^	NA	NA	6.39 × 10^−6^	Down	2.44 × 10^−7^	40	NA
pregnen-diol disulfate*	Steroid	Lipid	6.49 × 10^−6^	Down	3.06 × 10^−5^	15	NA
Cortisol	Steroid	Lipid	9.12 × 10^−6^	Down	4.40 × 10^−6^	29	NA
X – 17340 (tetrahydrocortisone glucuronide)^c^	NA	NA	9.15 × 10^−6^	Down	1.13 × 10^−4^	65	NA
16a-hydroxy DHEA 3-sulfate	Steroid	Lipid	9.46 × 10^−6^	Down	1.65 × 10^−4^	24	7
Cortisone	Steroid	Lipid	1.11 × 10^−5^	Down	6.35 × 10^−7^	25	NA
3-methyl-2-oxovalerate	Leucine, Isoleucine and Valine Metabolism	Amino Acid	1.30 × 10^−5^	Down	3.82 × 10^−2^	91	3
Prolylglycine	Dipeptide	Peptide	1.46 × 10^−5^	Up	NA	186	NA
etiocholanolone glucuronide	Steroid	Lipid	2.33 × 10^−5^	Down	6.58 × 10^−5^	18	NA
X – 21441	NA	NA	2.66 × 10^−5^	Down	3.16 × 10^−6^	22	NA
21-hydroxypregnenolone disulfate	Steroid	Lipid	3.11 × 10^−5^	Down	3.46 × 10^−3^	7	4
4-androsten-3alpha,17alpha-diol monosulfate (2)	Steroid	Lipid	3.31 × 10^−5^	Down	2.85 × 10^−6^	11	NA
pregnenolone sulfate	Steroid	Lipid	3.85 × 10^−5^	Down	3.62 × 10^−7^	10	NA

^a,b^Results from OPLS-DA analysis when considering all variables (all metabolites and all covariates), and after feature selection respectively.

^c^Tentative identification/renaming by Metabolon, and in some cases indicates same identification for two unknown metabolites.

*Indicates a compound that has not been officially confirmed by Metabolon based on a standard but it is confident of its identity.

**Figure 1:**
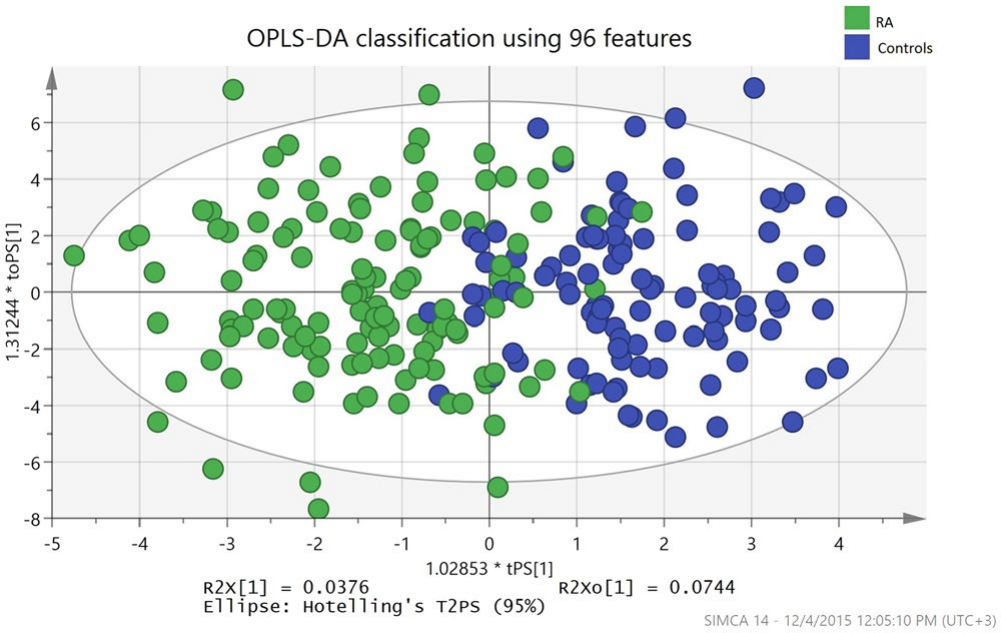
OPLS-DA prediction analysis results using 96 features (metabolites and covariates), sensitivity is 91.6% and specificity is 88.4%.

To investigate the robustness of using the identified metabolites to differentiate RA patients from controls, two experiments were conducted using OPLS-DA. The first experiment used all samples, all 927 metabolites and all covariates and resulted in 79.5% sensitivity, 75.9% specificity, with a p value (Fisher’s probability) of p = 4.5 × 10^−18^. After feature selection was done on metabolites and covariates (see Methods), a set of 94 metabolites and two covariates were used for OPLS-DA. This resulted in classification of RA samples with 91.6% sensitivity, and 88.4% specificity, with a p value (Fisher’s probability) of p = 7.1 × 10^−39^. Figure 1 shows the scatter diagram indicating the separation between RA patients and controls using OPLS-DA with the feature selection-based model. Discrimination of RA patients from controls is highly attributed to the 32 metabolites previously identified by linear regression. 22 out of the top 30 metabolites in the first experiment are in the identified set of 32 RA-associated metabolites with a Fisher’s exact p value of 0.022 (see Methods). Whereas, after feature selection, 6 out of the 7 highest ranked metabolites (7 top most RA discriminating variables) are in the list of the 32 RA associated metabolites. Table 2 shows the ranks of metabolites according to their ability to discriminate the two groups (see Supplemental Tables 2 and 3 for the output from OPLS-DA for the complete list of metabolites and covariates).

### Steroid levels among RA patients linked to corticosteroid treatment

We further analyzed the 32 RA metabolites in RA patients only to investigate the effect of covariates specific to RA on their metabolic profile. Feature selection was done (see Methods) for each metabolite to select which covariates are affecting its levels. Those covariates are: age, gender, diabetes, smoking, and batch, the presence of antigens (seropositive versus seronegative patients), duration of the disease, evidence of family history, corticosteroid treatment, biologics treatment, presence of autoimmune disease and presence of any of coronary/stroke or vascular disease. Regression of each metabolite while correcting for the selected covariates of that metabolite was then done. No significant associations where found between the metabolite levels and any of the covariates indicated except for corticosteroid treatment. 25 out of the 32 RA associated metabolites (mainly steroids) showed an association (p < 0.05/32) with corticosteroid treatment (see Table 2), with the lowest levels in those who were on corticosteroid treatment at the time of the study, and higher levels in those who never took corticosteroids, and the highest levels or which approach the levels of control group in those who received corticosteroid treatment in the past (see Supplemental Fig. 2).

We further computed the difference in metabolite levels between corticosteroid-treated and non-corticosteroid-treated patients as well as the difference in metabolite levels between currently corticosteroid-treated and past corticosteroid-treated patients. 24 of the total 25 metabolites associated with corticosteroid treatment, and one additional metabolite were found significantly lower in corticosteroid treated than in corticosteroid past-treated patients, compared to only 7 metabolites out of those 25 that were found significantly (p < 0.05/32) lower in corticosteroid treated than in non- corticosteroid treated patients (see Supplemental Table 4).

### Gaussian Graphical Modeling identifies 11 sub-networks that stratify RA associated metabolites

**Figure 2:**
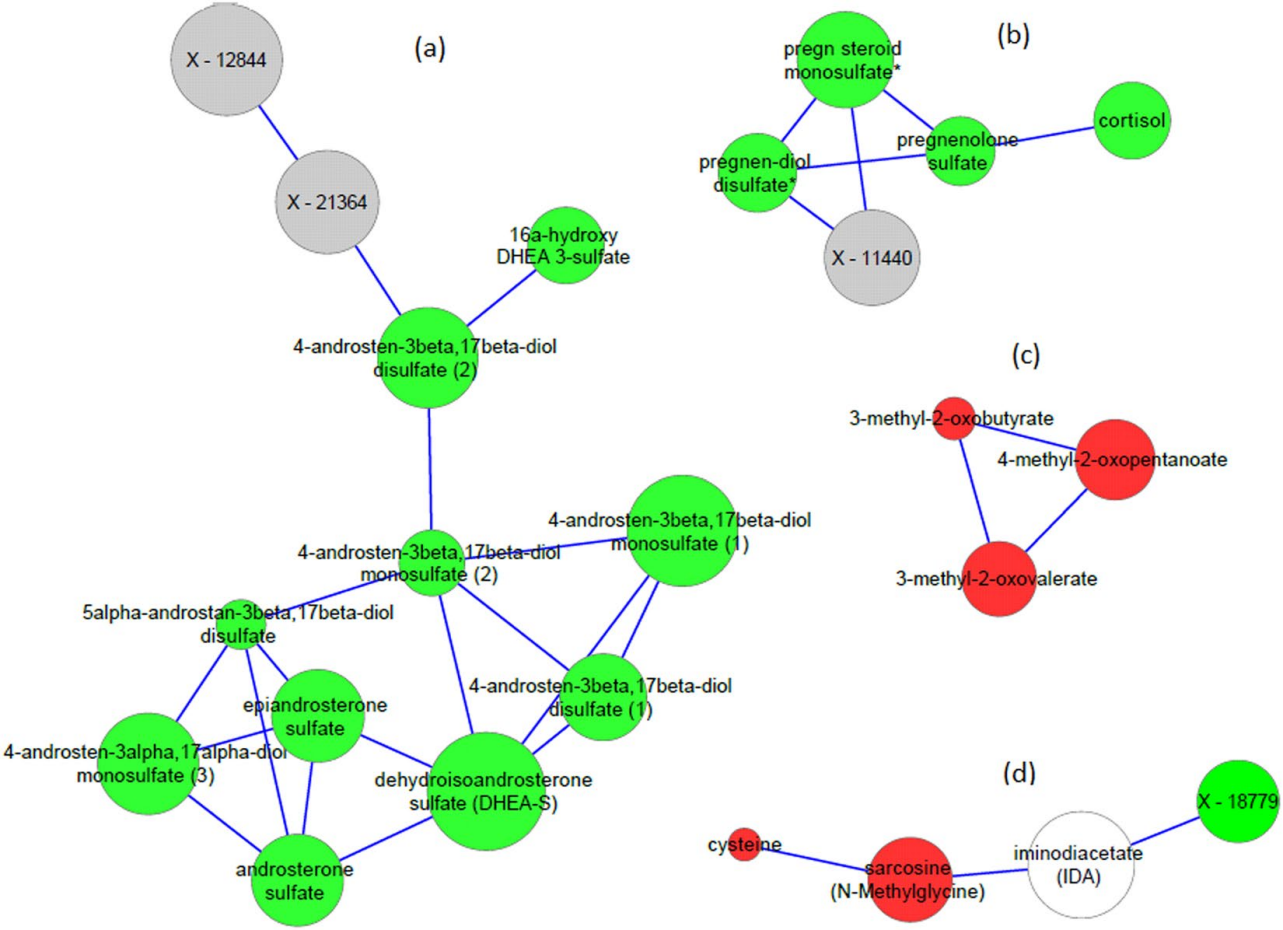
GGM sub-networks of metabolites that have a Bonferroni significant association with RA. Steroids sub-networks (**a**,**b**), BCAAs sub-network (**c**) and iminodiacetate sub-network (**d**). The size of the node proportional to – (log p value) (larger nodes indicate more significant association to RA). Red color indicates an amino acid, green indicates a lipid, white indicates a xenobiotic and grey indicates an unknown metabolite.

We computed pairwise partial correlations between all 927 metabolites, after correcting the data for RA, age, gender, smoking, batch effect and diabetes. 609 Bonferroni significant partial correlations (p <= 0.05/(927 * 926/2)) that span 554 metabolites were identified and used to build the GGM (nodes are metabolites and edges are significant partial correlations). Metabolites that were identified as significantly associated with RA, at a nominal significance of p < 0.05 were used to filter the GGM on sub-networks associated with RA (see Methods). As a result, a total of 11 sub-networks (containing more than 2 metabolites) were identified, 6 of which are formed of 3 metabolites, with the largest sub-network formed of 11 metabolites (see Supplemental Fig. 1). The four largest sub-networks - shown in Fig. 2 - are selected for further investigation.

12 out of the total 16 steroids that show Bonferroni significance appear in two sub-networks, the first with 8 Bonferroni significant steroids, and other 2 nominally significant steroids, and other 2 unknowns: X-12844 and X-11440 (interestingly these are tentatively identified by Metabolon as tetrahydrocortisone glucuronide and hydroxypregnen-diol disulfate (or pregnenolone-diol disulfate) respectively), and the second sub-network with 4 steroids and one unknown. The first sub-network connects epiandrosterone sulfate, dehydroisoandrosterone sulfate (DHEA-S) and androsterone sulfate together, then connects each to an androstenediol (beta or alpha) metabolite where there are 6 metabolites of this family in this network, i.e. comprising the largest part of the sub-network. The steroids in the second largest sub-network are metabolites from the pregn steroid family, namely: pregn, pregnen and pregnenolone sulfate or disulfate metabolites connected to cortisol, and X – 11440 (tentatively identified as hydroxypregnen-diol disulfate (or pregnenolone-diol disulfate)).

Non-steroid metabolites that are Bonferroni significant are also grouped into 2 sub-networks; one is formed of 3 metabolites in the BCAAs pathway, namely 4-methyl-2-oxopentanoate and 3-methyl-2-oxovalerate and another nominally significant metabolite 3-methyl-2-oxobutyrate. The second sub-network connects the xenobiotic (iminodiacetate), and the glycine-serine-threonine metabolite (sarcosine) to X – 18779 and the nominally significant metabolite cysteine.

## Discussion

Using non-targeted metabolomics approach, we identified 32 metabolites to be associated with established RA as compared to controls, with an enrichment in the steroid pathway. We showed that the 32 RA associated metabolites play a role in discriminating RA samples from controls. We also found that patients who were on corticosteroid treatment at the time of the study had the lowest steroid levels, compared to patients who received corticosteroid treatment in the past or those who never did. Regarding GGMs, we showed that four GGM sub-networks relate to steroidogenesis and amino acid metabolism/synthesis. In alignment with steroidogenesis, one network relates the precursor steroid pregnenolone to cortisol (Fig. 2b), while a second network (Fig. 2a) relates the sulfated forms of the adrenal androgens, DHEA, androstenedione, androstenediol, androsterone and epiandrosterone and their respective alpha and beta isomers^15–17^. A third  sub-network relates to transaminase metabolites of BCAAs and the fourth sub-network refers to cysteine and N-methylglycine metabolism.

Our results suggest deficiency of a larger number of steroids in RA patients compared to what was previously observed in literature. Previous research has established that the hypothalamic pituitary adrenocortical-axis (HPA), hypothalamic pituitary gonadal-axis (HPG) and the sympathetic nervous system (SNS) are all perturbed in rheumatoid arthritis^18–21^. Although inflammation (via IL-6 and TNF) normally stimulates the HPA axis during acute stress, chronic inflammation, as in RA, appears to suppress the normal function of HPA^22–24^, resulting in decreased levels of DHEA-S and inappropriately normal or low cortisol relative to the level of inflammation^21, 25, 26^. *In vitro* studies have supported this view by demonstrating an inhibitory effect of inflammatory cytokines on adrenal steroidogenesis enzymes P450c17, P450scc, P450c21 P450c11 and the sulfation mechanism of DHEA^15, 16^. In addition, DHEAS was observed to be decreased in RA patients^18, 27^, in pre-RA patients^28^ and in premenopausal women with RA^29^ as well as in self-reported joint inflammation^30^, compared to controls. Perturbations in DHEAS levels were also linked to severity^31^ and duration of RA^18^. Moreover, anti-TNF agents used to treat RA have shown a reversal of these suppressive effects including an increase in DHEAS, improved physical functioning^32^ and improved HPA response. As previously mentioned in^30^, ^33^, the unknown metabolites X-12844 (tentatively identified as tetrahydrocortisone glucuronide), X-11440 (tentatively identified as hydroxypregnen-diol disulfate (or pregnenolone-diol disulfate)) and X-11444 (tentatively identified as urocortisol glucuronide or cortolone glucuronide) have also been previously reported to be associated with genetic variants related to steroid metabolism. For example, X-11440 has been found to be associated with a SNP near SULT2A1 which catalyzes the sulfation of steroids, particularly preferring the sulfation of DHEA. Other steroids that we found to be decreased in RA include 16aOH-DHEAS and pregnenolone sulfate. 16a-OH DHEA is formed by the action of non-specific CYP450 monooxygenases on DHEA but its functions are poorly understood^34, 35^. The conversion of DHEA to 16a-OH DHEA and its aromatization to 16aOH-estrogens has been demonstrated in synovial cells of patients with RA and osteoarthritis^36^. It may be hypothesized that 16aOH-DHEA is decreased due to lower conversion of DHEAS to DHEA in the synovium of patients with rheumatoid arthritis or due to the depletion of this precursor in the formation of 16a- estrogens, due to increased aromatase activity^37, 38^. Pregnenolone, which acts as the initial metabolite of the StAR P450scc rate-limiting step of adrenal steroidogenesis, is also decreased perhaps reflecting the influence of inflammation on this step of steroidogenesis^39^. Overall, our results support previous findings that suggest deficiency of several adrenal steroids in RA patients, and extends those findings to a larger number of steroids, further highlighting perturbations in the steroidogenesis pathway in RA.

The RA-associated non-steroid metabolites (4-methyl-2-oxopentanoate, 3-methyl-2-oxovalerate, sarcosine and prolylglycine) were all decreased in patients, except for prolylglycine which was higher in patients compared to controls. Perturbations in BCAAs metabolism, namely decreased 4-methyl-2-oxopentanoate and 3-methyl-2-oxovalerate (among the first step catabolites of BCAAs) levels in patients, is in line with what was observed in late stage osteoarthritis patients^8^, knowing that decreased amino acids levels are associated with cartilage destruction.

We evaluated the effect of corticosteroid treatment on the metabolites and found that levels of steroids in past corticosteroid-treated patients are higher than those who were on corticosteroid treatment and those who never took the treatment. All steroid metabolites that showed association to corticosteroid treatment – except for one metabolite - also showed significant increase in past corticosteroid-treated patients compared to corticosteroid-treated ones. On the other hand, only 7 metabolites showed significant increase in patients who have never been corticosteroid-treated compared to corticosteroid-treated patients (see Supplemental Table 4 and Supplemental Fig. 2 for results and boxplots of steroid levels in all 3 groups of corticosteroid treatment). Patients with no corticosteroid-treatment had lower steroid levels than those who had past corticosteroid treatment and higher levels than those who were corticosteroid-treated. Such decrease in steroids among RA patients receiving corticosteroid may be due to the suppressive effects of exogenous steroids on the HPA. These effects on the metabolome have been described in healthy male volunteers, resulting in a significant decrease in the levels of DHEAS, cortisol, corticosterone, and androstenedione^40^. The decreased steroids in those who had never received corticosteroid may be revealing an inherent steroid deficiency in patients with RA, which is further pronounced by exogenous corticosteroids. Those findings could alternatively be explained by a subgroup of patients with RA who is more prone to steroid “deficiency” and to be steroid dependent.

### Limitations of the study

Our study has several limitations as follows: 1) patients and controls were not matched for age, gender, diabetes, smoking and batch effect, yet adjusting for these covariates in the regression model gives equivalent statistical power to using matched samples^41, 42^. We have also computed the correlation of each of age, gender, diabetes and smoking to batch effect (Supplemental Table 5), to ensure that there is no bias in one of the batches for those phenotypes. 2) There was no fasting/non-fasting condition specified prior to sample collection, yet most of the participants did not have a major meal at least 2 hours prior to sampling which we believe is enough to reduce any effect on the metabolic profile related to food intake. Moreover, samples were collected as they became available at the same location, in a random pattern using identical protocols, instruments and study personnel. 3) BMI was not included as a covariate as it was not available for all samples, however the obtained RA associated metabolites results did not include known BMI associated metabolites^43^. 4) Disease severity, activity measures, or intake of any other supplements could not be accounted for when doing comparisons among the RA groups. However, by accounting for the various factors that impact the RA patients, specifically the presence of autoimmune, vascular diseases, family history, duration of the disease, treatment with Biologics and DMARDs, we have corrected for most of variations that would affect their metabolic profiles. Those limitations might have increased a random error in the data, yet would not create any spurious signals. Having detected 32 metabolites associated with RA under these conditions, and with replication of previous results, shows the robustness of our findings.

## Conclusion

We identified 32 RA-associated metabolites in a cohort of 236 samples with an enrichment in the steroid pathway, thus emphasizing on the perturbations of the hypothalamic pituitary adrenocortical-axis (HPA) and neuroendocrine system. Our results replicate the previously known metabolites as DHEAS, cortisol and androstenedione, yet highlights the role of a larger number of steroids in the pathomechanism of RA. OPLS-DA classification of RA samples with a sensitivity of 91% and specificity of 88%, supports the evidence that the identified metabolites are perturbed in RA patients. The GGM modeling reveals correlations between metabolites similar to those of the steroidogenesis pathway. Our study is the first to report a large number of steroids involved in RA pathomechanism due to the use of a new platform by Metabolon untargeted technology, and a larger number of samples (236) compared to previous studies. Our study also showed that past corticosteroid-treated patients show higher levels of steroids than any of the currently corticosteroid-treated or non-corticosteroid-treated patients, and that corticosteroid-treated patients have lower steroid levels than non-corticosteroid-treated patients. Whether it is the effect of the treatment or the severity of the disease that caused such low steroid levels in corticosteroid-treated patients is still arguable. Future studies will be needed to discover the effect of those factors on metabolite levels in larger samples of RA patients, with more information on disease activity and the dose of the medication.

## Electronic supplementary material

Supplemental Information

Supplemental Table 1

Supplemental Table 2

Supplemental Table 3

Supplemental Table 4

Supplemental Table 5
