# Supplementary material for: Large Scale Metabolic Profiling identifies Novel Steroids linked to Rheumatoid Arthritis
Source: Sci Rep. 2017 Aug 22;7:9137. doi: 10.1038/s41598-017-05439-1 (PMC5567269; doi:10.1038/s41598-017-05439-1)
Supplement: Supplementary file 1 — Supplemental Information [file 41598_2017_5439_MOESM1_ESM.pdf]

Large Scale Metabolic Profiling identifies Novel Steroids linked to Rheumatoid Arthritis

Noha A. Yousri[1,2], Karim Bayoumy[3,4], Wessam Gad Elhaq[3,14], Robert P. Mohney[5],Samar Al Emadi[6,15], Mohammed Hammoudeh[6,15], Hussein Halabi [7,15], Basel Masri [8,15], Humeira Badsha[9,15], Imad Uthman[10,15], Robert Plenge[11,15], Richa Saxena[12,13, 15], Karsten Suhre[1], Thurayya Arayssi[14,15]

[1]Bioinformatics Core, Physiology and Biophysics, Weill Cornell Medicine- Qatar, Doha. [2]Computers and Systems Engineering, Faculty of Engineering, Alexandria University, Egypt. [3]Research Division, Weill Cornell Medical College-Qatar, Education City, Doha, Qatar.[4]Department of Internal Medicine, Hamad Medical Corporation,Doha,Qatar.[5]Metabolon Inc., Durham, NC, USA.[6]Division of Rheumatology, Department of Medicine, Hamad Medical Corporation, Doha, Qatar.[7]King Faisal Specialist Hospital & Research center, Jeddah, Kingdom of Saudi Arabia.[8]Jordan Hospital, Amman, Jordan.[9]Dr. Humeira Badsha Medical Center, Dubai, UAE.[10]American University of Beirut, Faculty of Medicine, Beirut, Lebanon.[11]Merck, Boston, MA, USA, Division of Rheumatology, Immunology, and Allergy, Brigham and Women's Hospital, Harvard Medical School, Boston, MA, USA.[12]Center for Human Genetic Research, Department of Anesthesia, Critical Care and Pain Medicine, Massachusetts General Hospital, Harvard Medical School, Boston, MA, USA.[13]Program in Medical and Population Genetics, Broad Institute, Cambridge, MA, USA.[14]Department of Medicine, Weill Cornell Medical College-Qatar, Education City, Doha, Qatar.[15]Member of the Middle East Rheumatoid Arthritis Consortium (MERAC)

Supplemental Information:

Supplemental Figure 1: GGM subnetworks where nodes correspond to metabolites and edges are significant partial correlations. Size of the node is proportional to its significance of association with RA. The color of the node indicates the superpathway of the metabolite according to the legend.

Supplemental Figure 2: Boxplots of 32 metabolites associated with RA for each of the RA groups with different corticosteroid treatment.

Supplemental Methods: Interaction Model.

Supplemental Table 1: Results of regression of all metabolites against case/control while including covariates and using interaction model.

Supplemental Table 2: Output from OPLS-DA analysis using 96 metabolites and covariates.

Supplemental Table 3: SIMCA OPLS-DA output using all features (metabolites and covariates).

Supplemental Table 4: Results of regression of 32 RA metabolites in RA samples only using covariates according to feature selection.

Supplemental Table 5: Correlation of batch (KSA batch and Qatar batch) with different phenotypes.

Supplemental Figure 1: GGM subnetworks where nodes correspond to metabolites and edges are significant partial correlations. Size of the node is proportional to its significance of association with RA. The color of the node indicates the superpathway of the metabolite according to the legend.

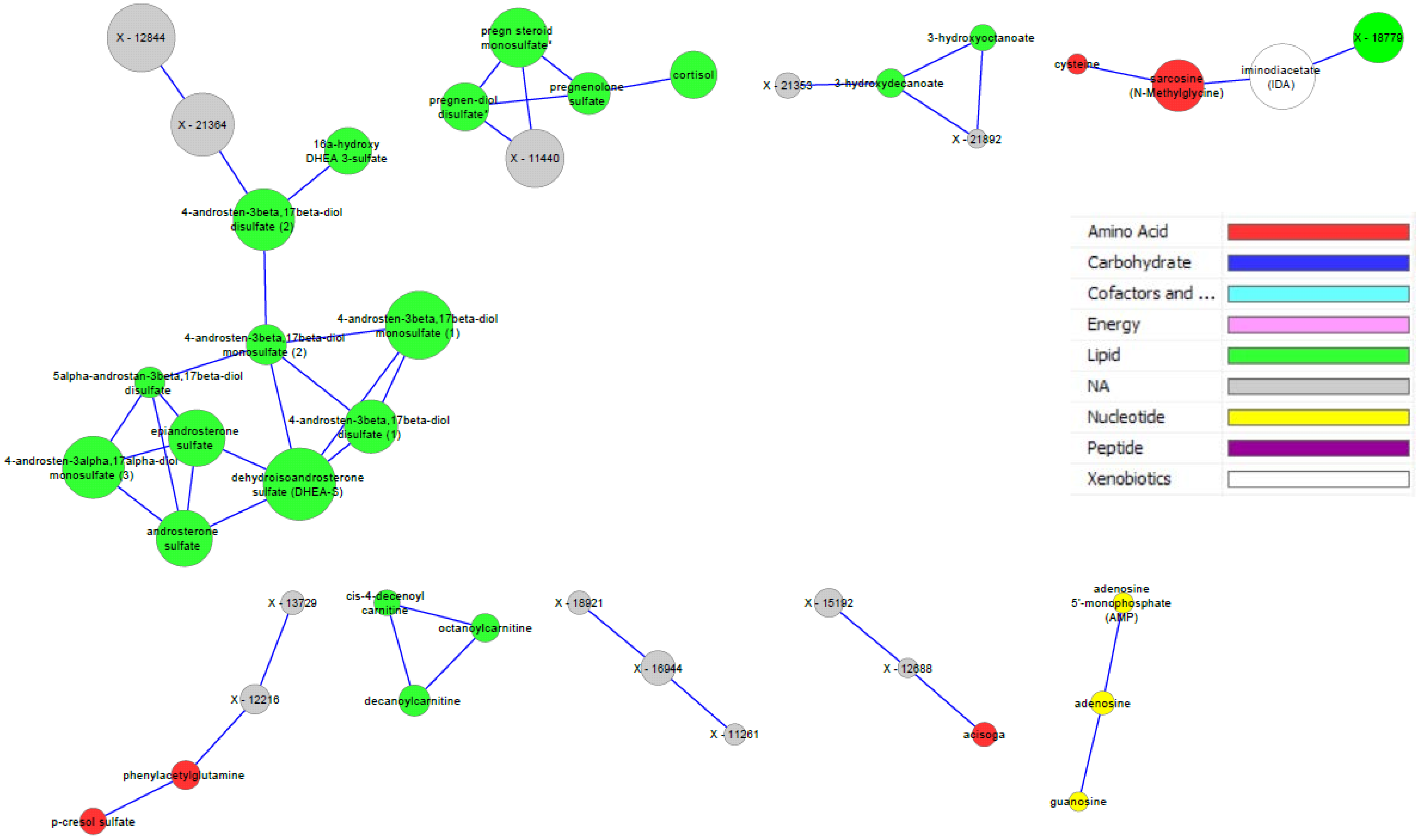
X - 12844
X - 21364
16a-hydroxy DHEA 3-sulfate
4-androsten-3beta,17beta-diol disulfate (2)
4-androsten-3beta,17beta-diol monosulfate (2)
4-androsten-3beta,17beta-diol monosulfate (1)
5alpha-androstan-3beta,17beta-diol disulfate
epiandrosterone sulfate
4-androsten-3beta,17beta-diol disulfate (1)
4-androsten-3alpha,17alpha-diol monosulfate (3)
dehydroisoandrosterone sulfate (DHEA-S)
androsterone sulfate
pregn steroid monosulfate*
pregnen-diol disulfate*
pregnenolone sulfate
cortisol
X - 11440
3-hydroxyoctanoate
X - 21353
3-hydroxydecanoate
X - 21892
cysteine
sarcosine (N-Methylglycine)
iminodiacetate (IDA)
X - 18779
Amino Acid
Carbohydrate
Cofactors and ...
Energy
Lipid
NA
Nucleotide
Peptide
Xenobiotics
X - 13729
X - 12216
phenylacetylglutamine
p-cresol sulfate
cis-4-decenoyl carnitine
octanoylcarnitine
decanoylcarnitine
X - 18921
X - 16944
X - 11261
X - 15192
X - 12688
acisoga
adenosine 5'-monophosphate (AMP)
adenosine
guanosine

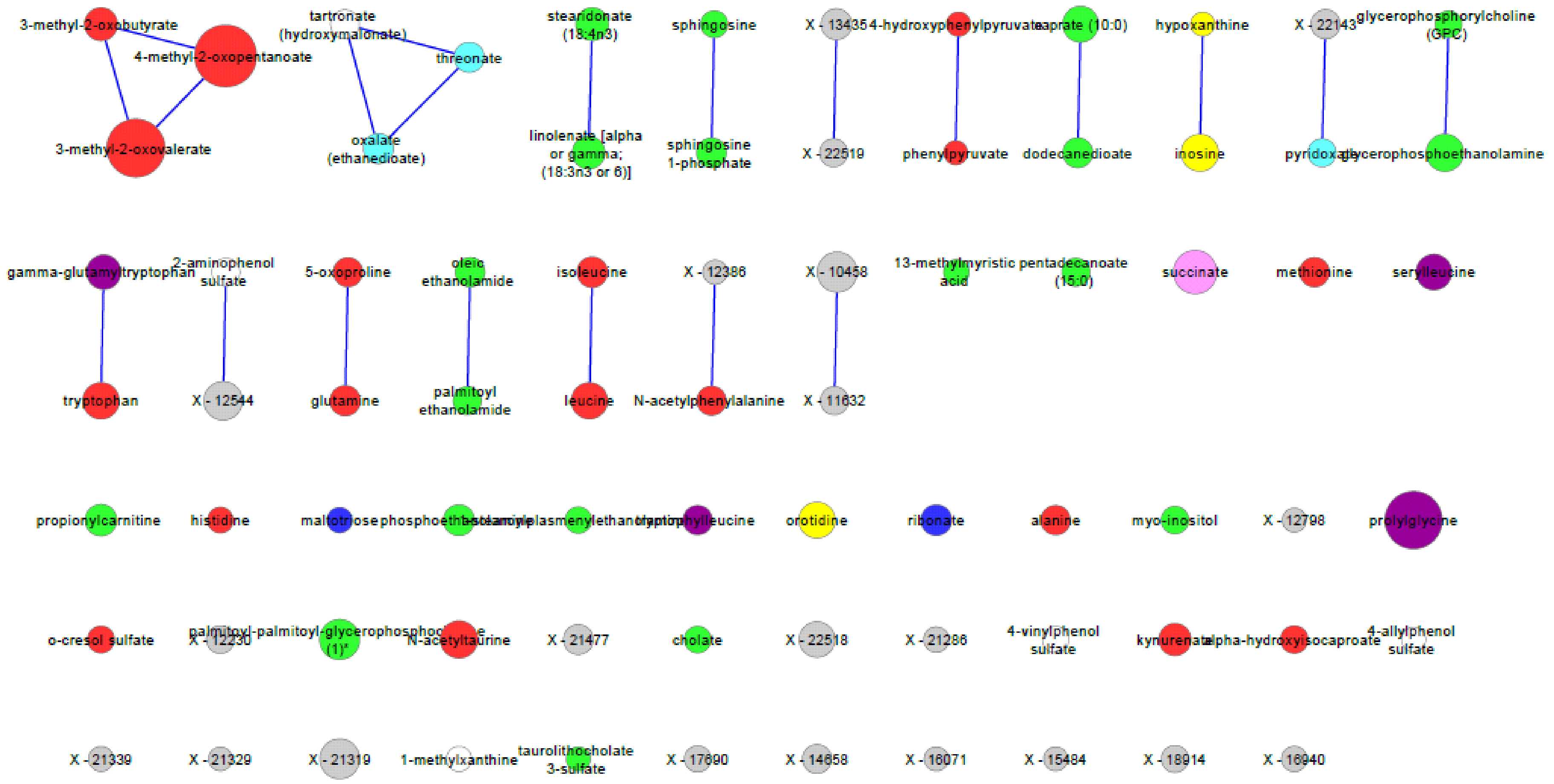
3-methyl-2-oxobutyrate
4-methyl-2-oxopentanoate
3-methyl-2-oxovalerate
tartronate (hydroxymalonate)
threonate
oxalate (ethanedioate)
stearidonate (18:4n3)
linolenate [alpha or gamma; (18:3n3 or 6)]
sphingosine
sphingosine 1-phosphate
X - 13435
X - 22519
4-hydroxyphenylpyruvate
phenylpyruvate
caprate (10:0)
dodecanedioate
hypoxanthine
inosine
X - 22143
pyridoxate
glycerophosphorylcholine (GPC)
glycerophosphoethanolamine
gamma-glutamyltryptophan
tryptophan
2-aminophenol sulfate
X - 12544
5-oxoproline
glutamine
oleic ethanolamide
palmitoyl ethanolamide
isoleucine
leucine
X - 12386
N-acetylphenylalanine
X - 10458
X - 11632
13-methylmyristic acid
pentadecanoate (15:0)
succinate
methionine
serylleucine
propionylcarnitine
histidine
maltotriose
phosphoethanolamine
1-stearoylplasmenylethanolamine
tryptophylleucine
orotidine
ribonate
alanine
myo-inositol
X - 12798
prolylglycine
o-cresol sulfate
X - 12230
palmitoyl-palmitoyl-glycerophosphocholine (1)*
N-acetyltaurine
X - 21477
cholate
X - 22518
X - 21286
4-vinylphenol sulfate
kynurenate
alpha-hydroxyisocaproate
4-allylphenol sulfate
X - 21339
X - 21329
X - 21319
1-methylxanthine
taurolithocholate 3-sulfate
X - 17690
X - 14658
X - 18071
X - 15484
X - 18914
X - 16940

Supplemental Figure 2: Boxplots of 32 metabolites associated with RA for each of the RA groups with different corticosteroid treatment.

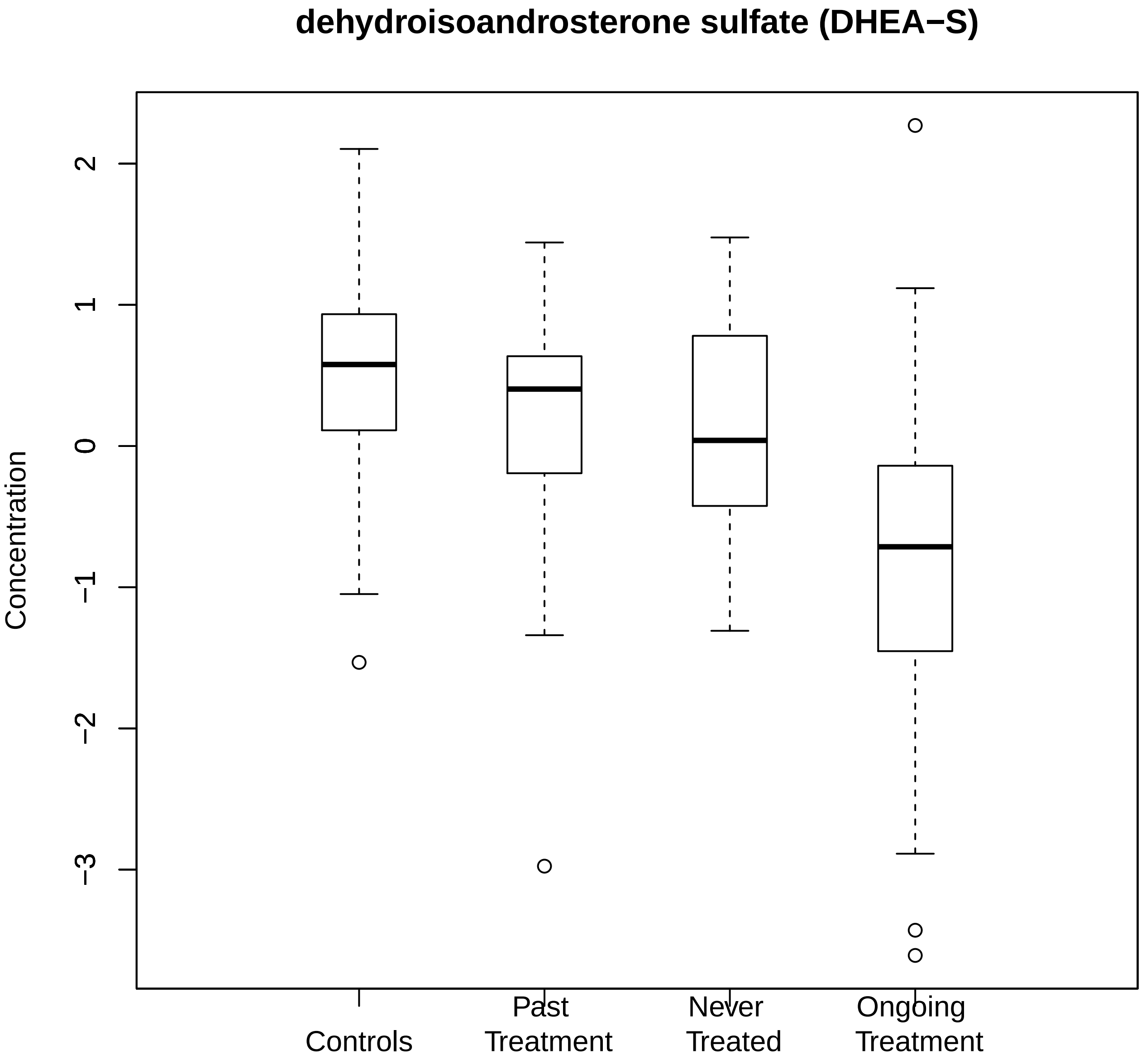

dehydroisoandrosterone sulfate (DHEA−S)
Concentration
2
1
0
−1
−2
−3
Controls
Past Treatment
Never Treated
Ongoing Treatment

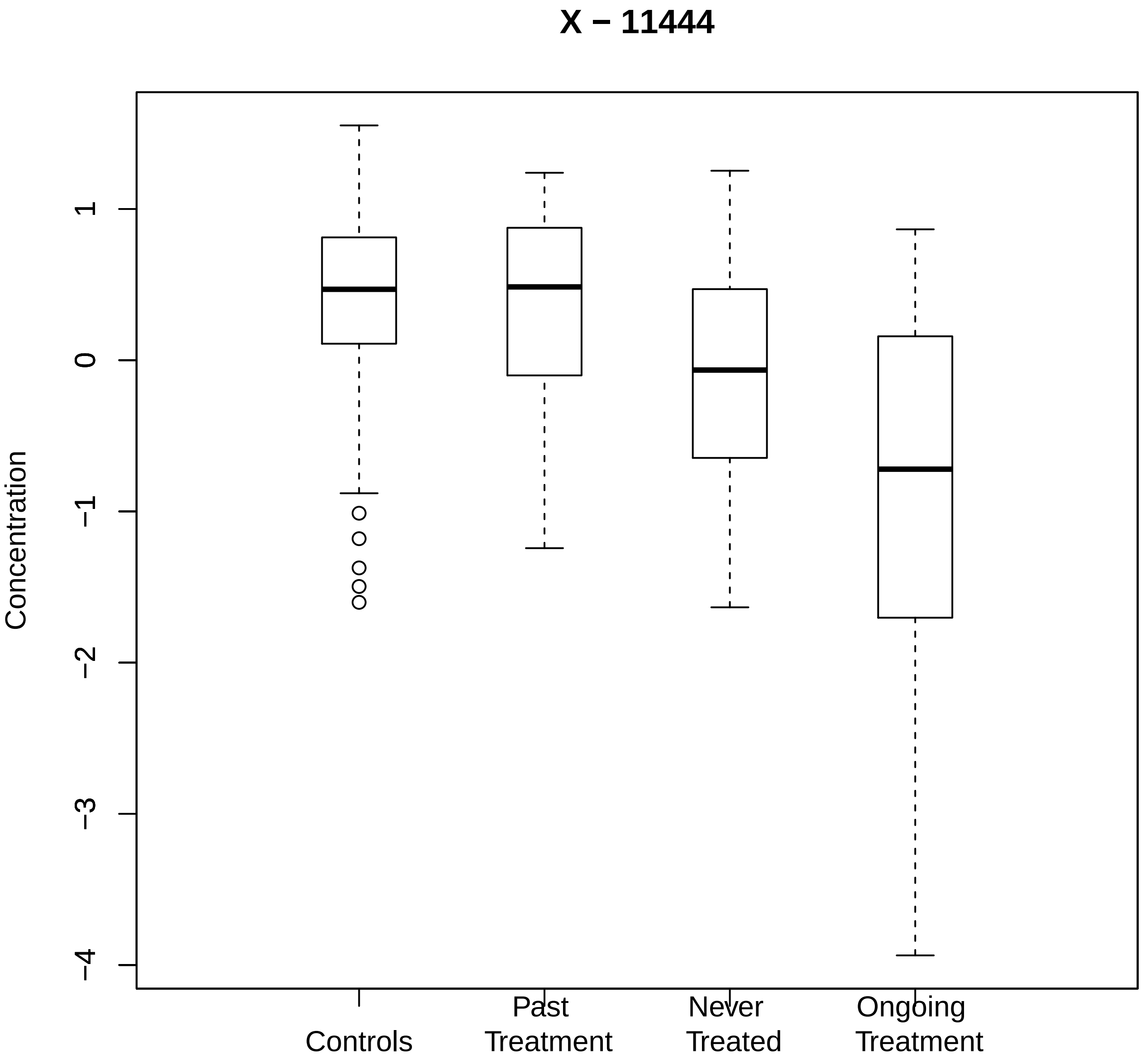
X − 11444
Concentration
1
0
−1
−2
−3
−4
Controls
Past Treatment
Never Treated
Ongoing Treatment

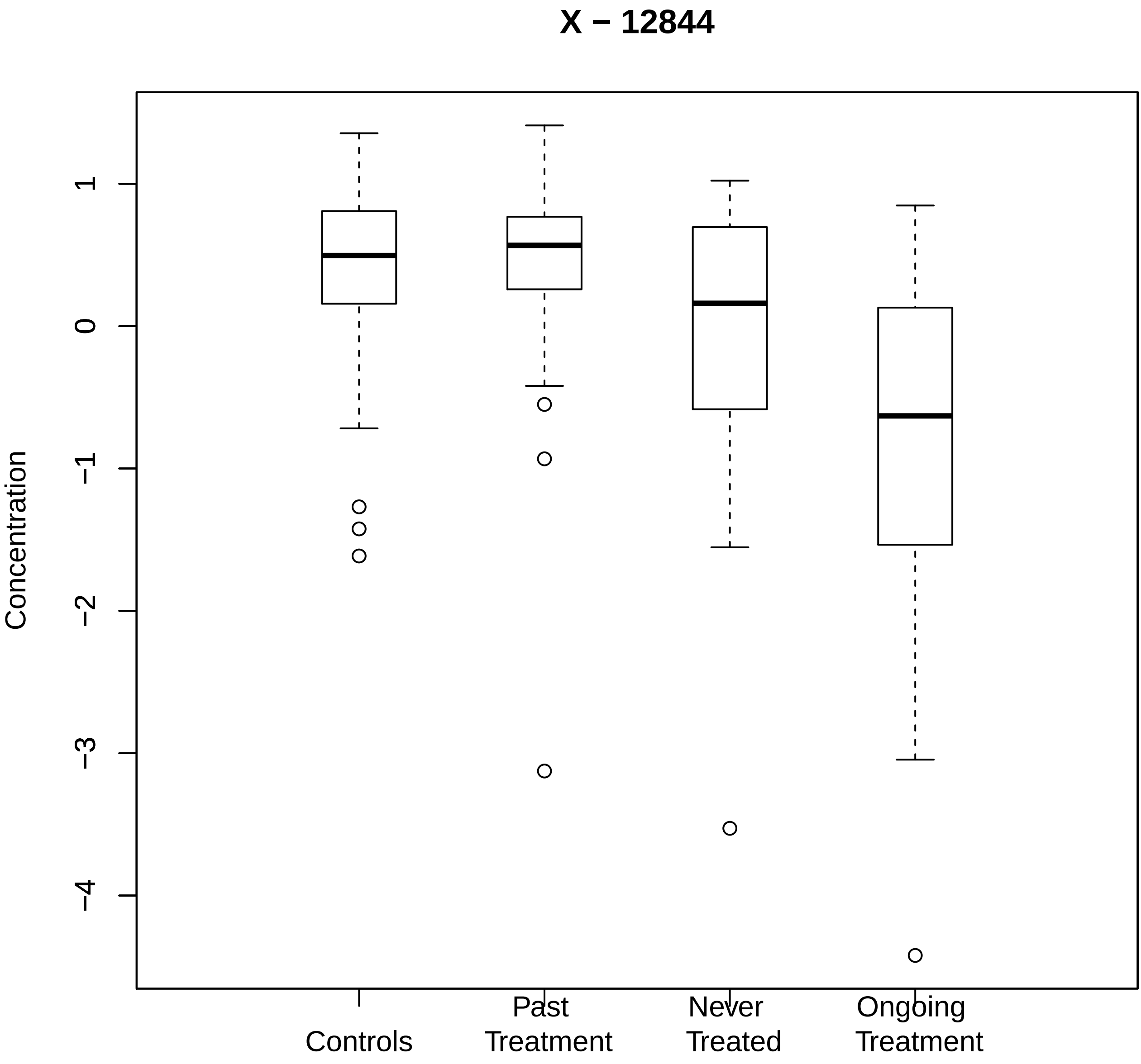

X − 12844
Concentration
1
0
−1
−2
−3
−4
Controls
Past Treatment
Never Treated
Ongoing Treatment

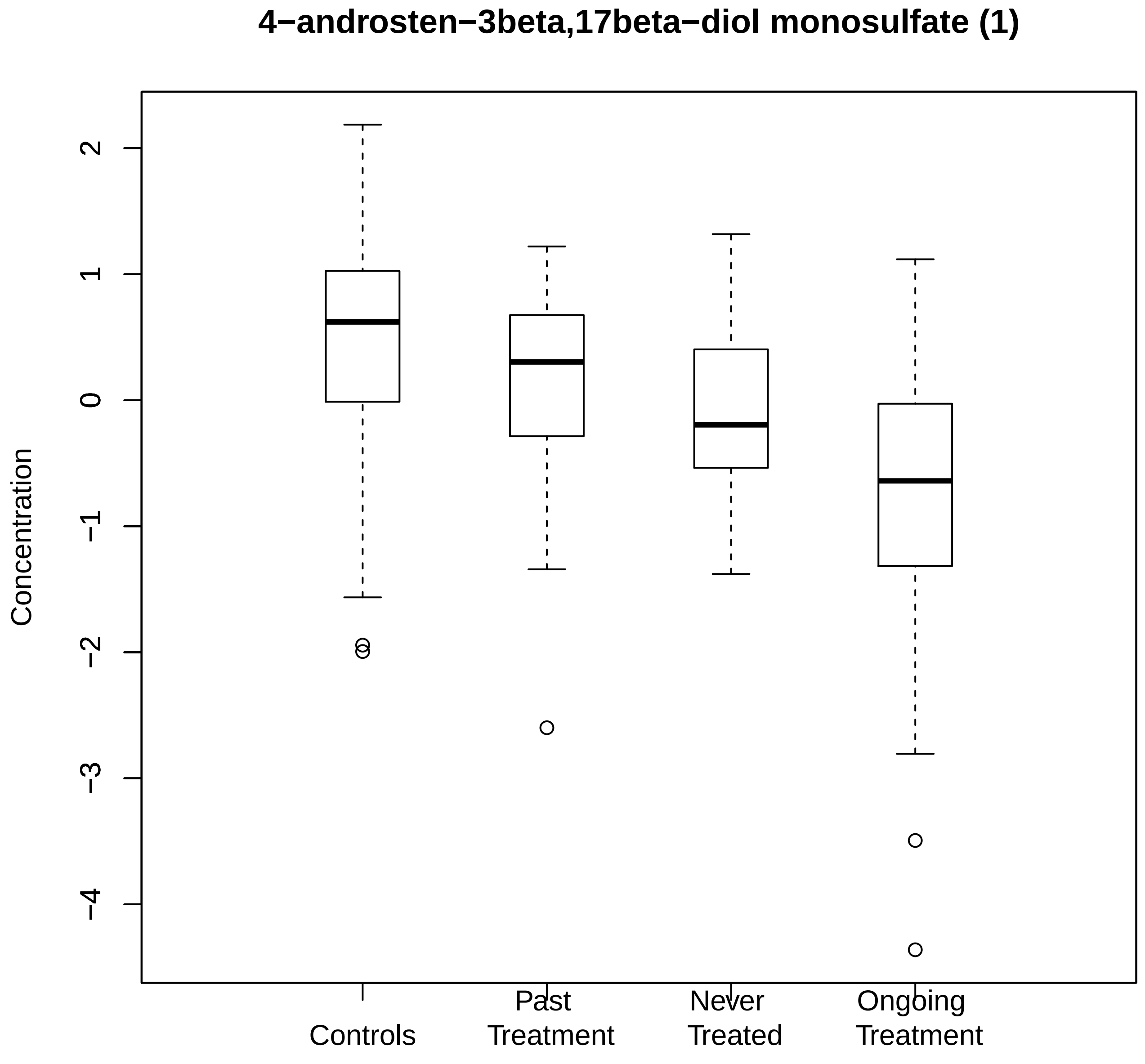

4−androsten−3beta,17beta−diol monosulfate (1)
Concentration
2
1
0
−1
−2
−3
−4
Controls
Past Treatment
Never Treated
Ongoing Treatment

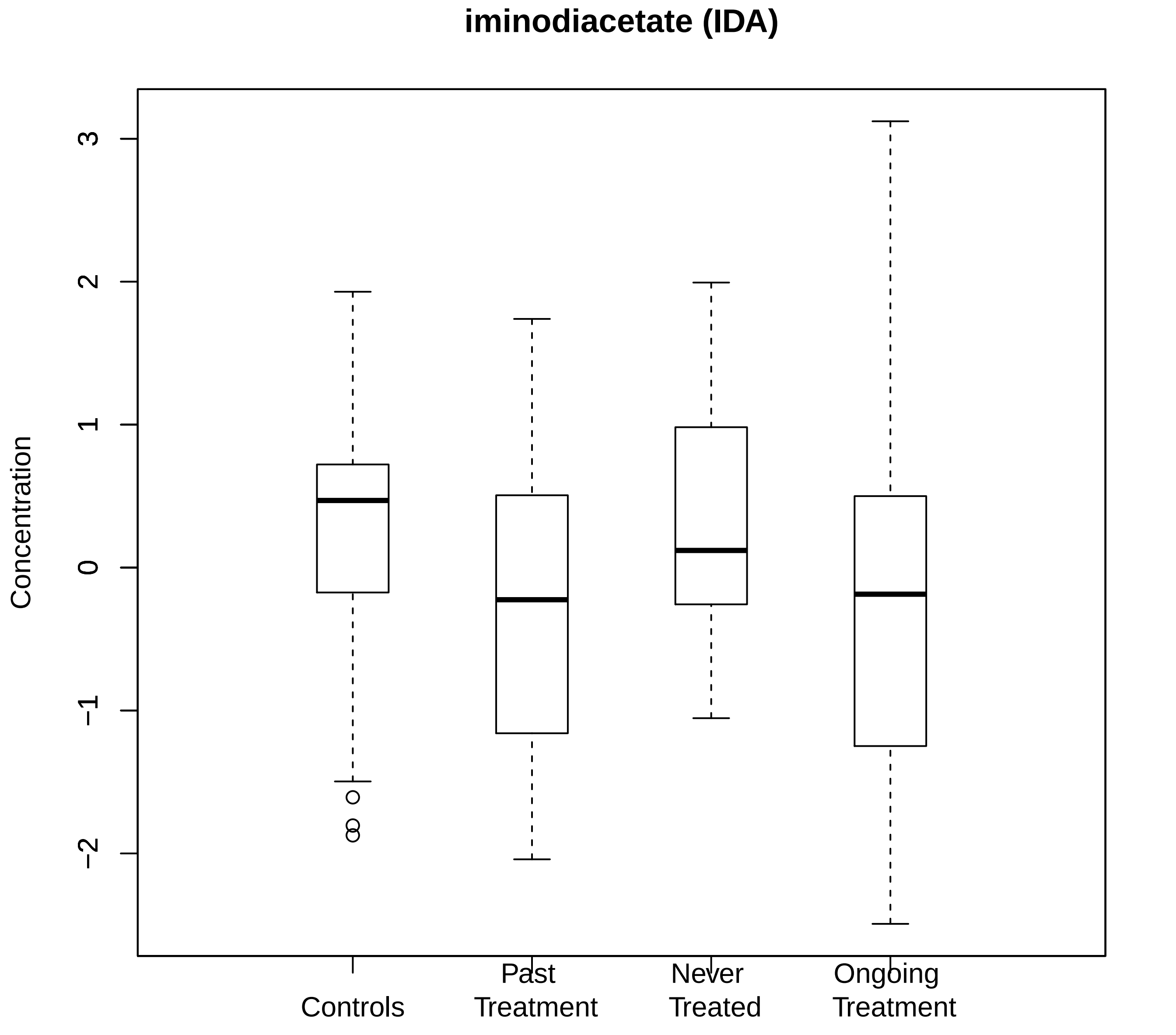
iminodiacetate (IDA)
Concentration
3
2
1
0
−1
−2
Controls
Past Treatment
Never Treated
Ongoing Treatment

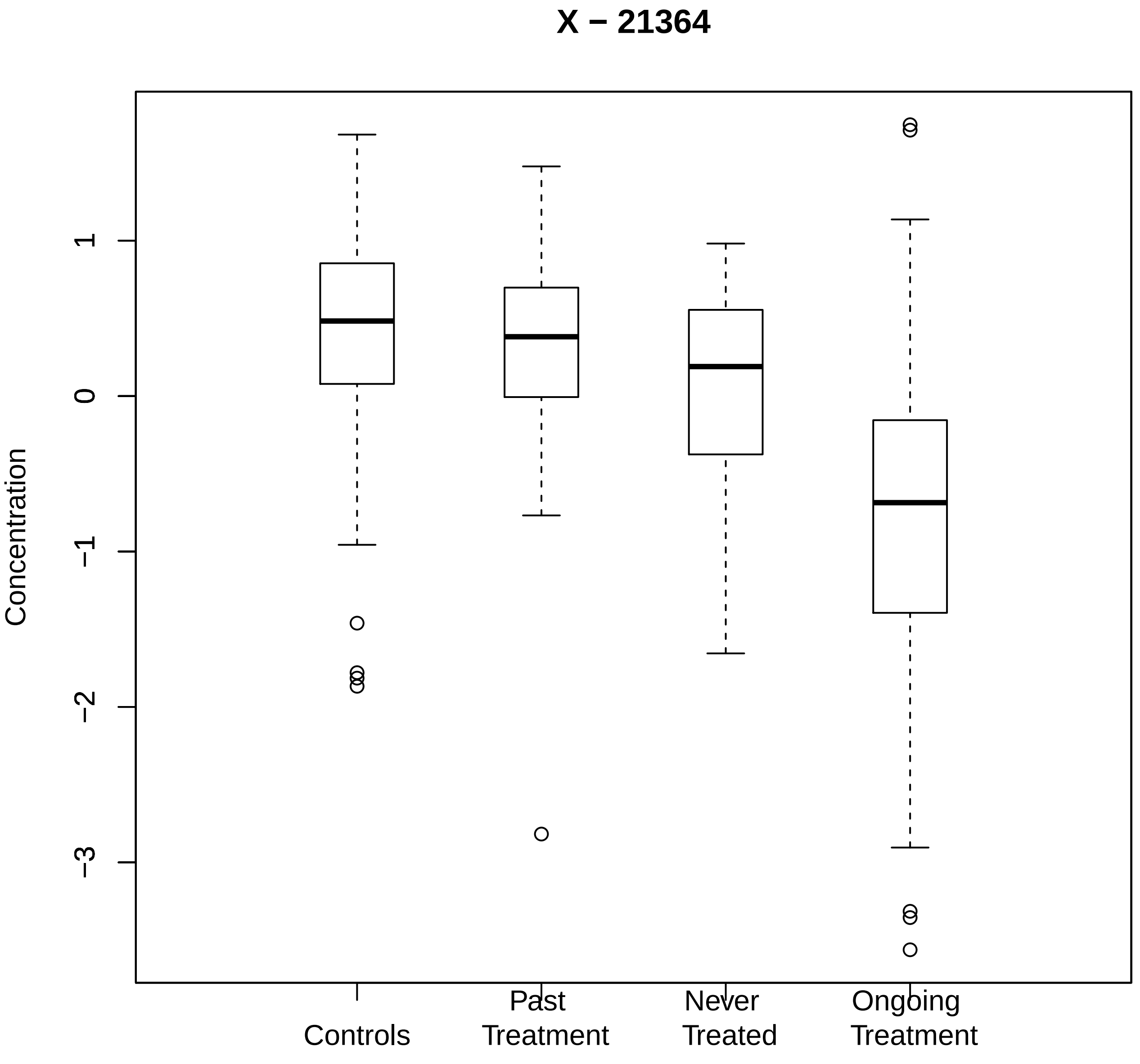

X − 21364
Concentration
1
0
−1
−2
−3
Controls
Past
Treatment
Never
Treated
Ongoing
Treatment

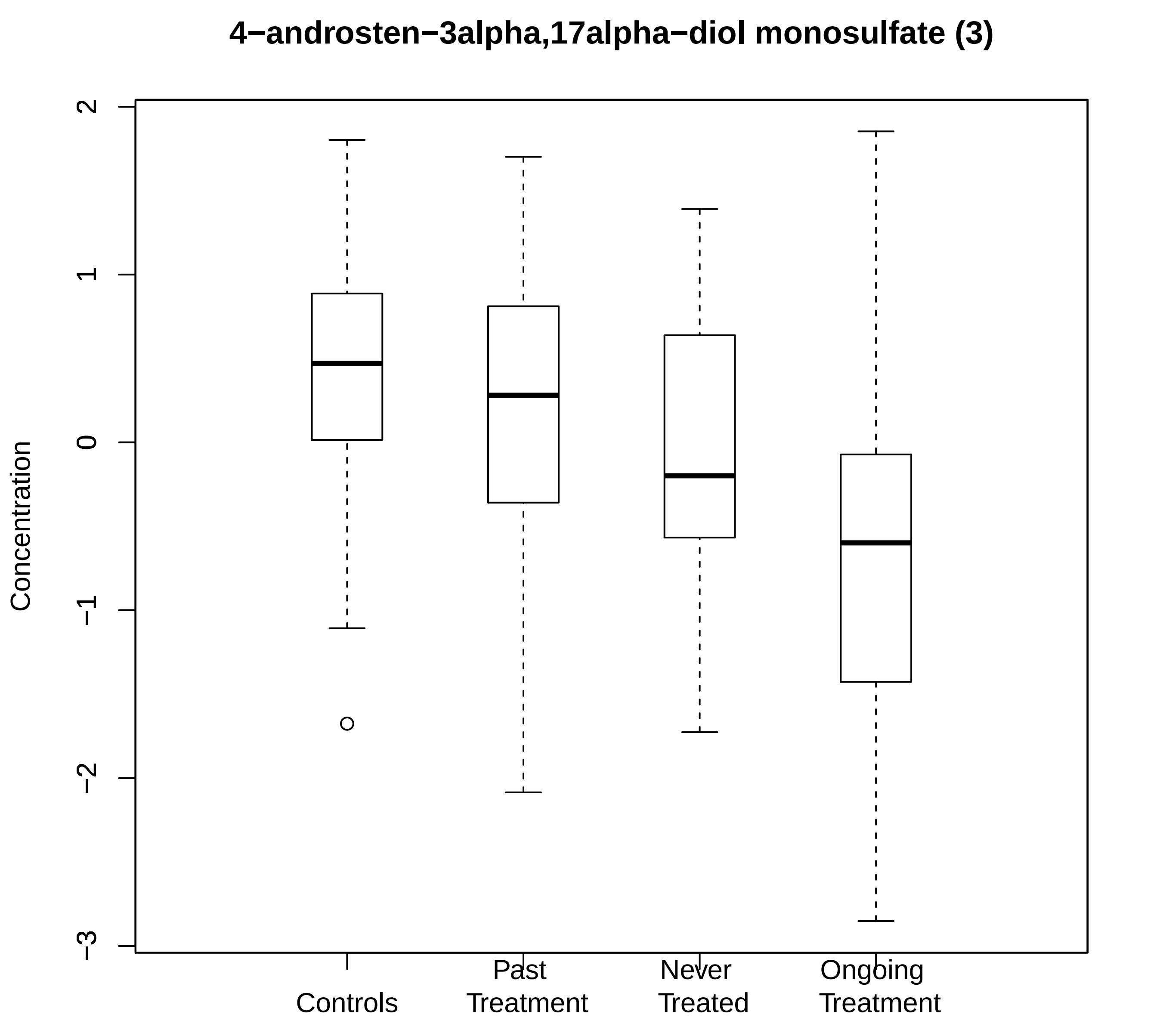

4−androsten−3alpha,17alpha−diol monosulfate (3)
Concentration
2
1
0
−1
−2
−3
Controls
Past
Treatment
Never
Treated
Ongoing
Treatment

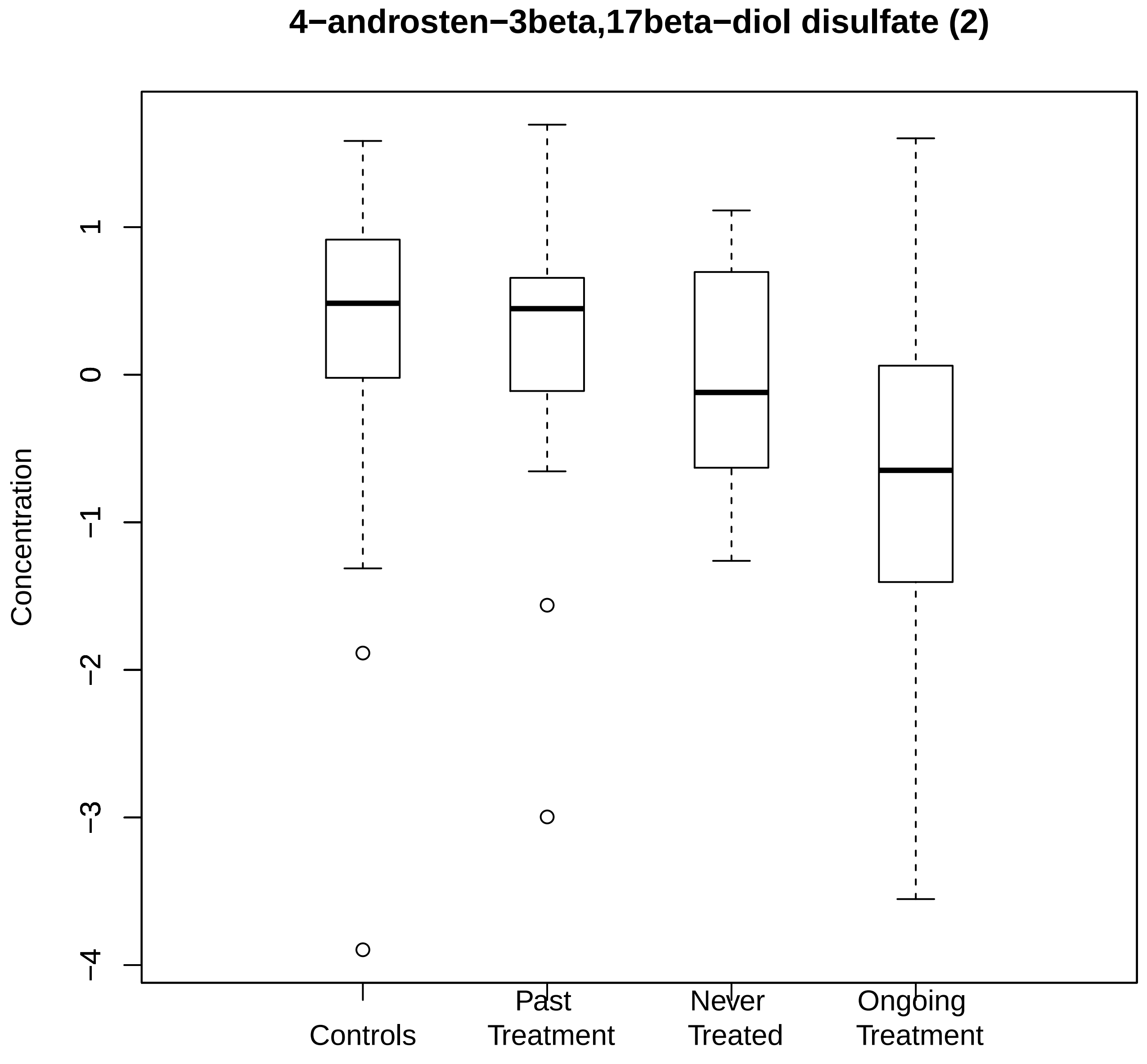
4−androsten−3beta,17beta−diol disulfate (2)
Concentration
1
0
−1
−2
−3
−4
Controls
Past Treatment
Never Treated
Ongoing Treatment

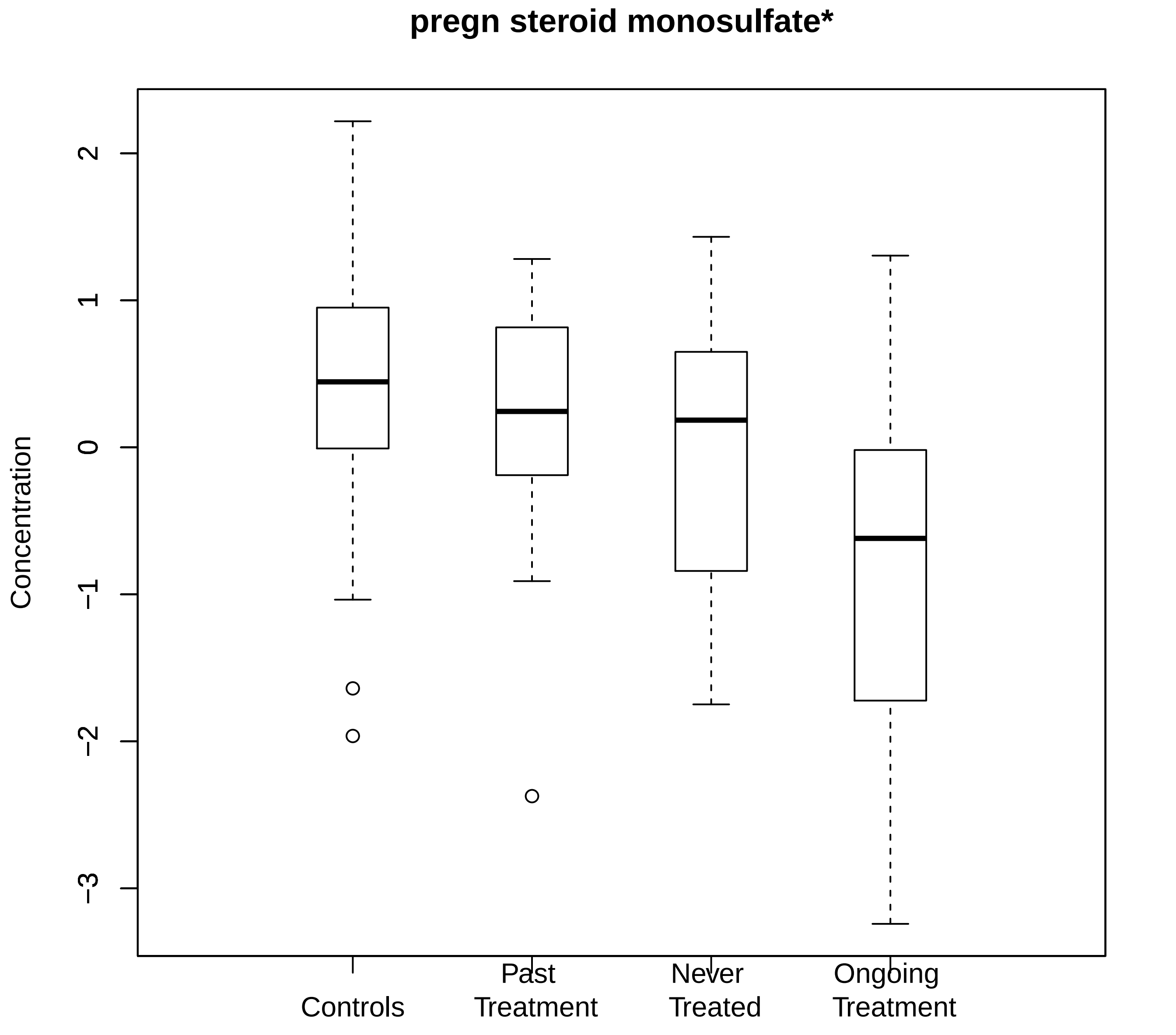
pregn steroid monosulfate*
Concentration
2
1
0
−1
−2
−3
Controls
Past Treatment
Never Treated
Ongoing Treatment

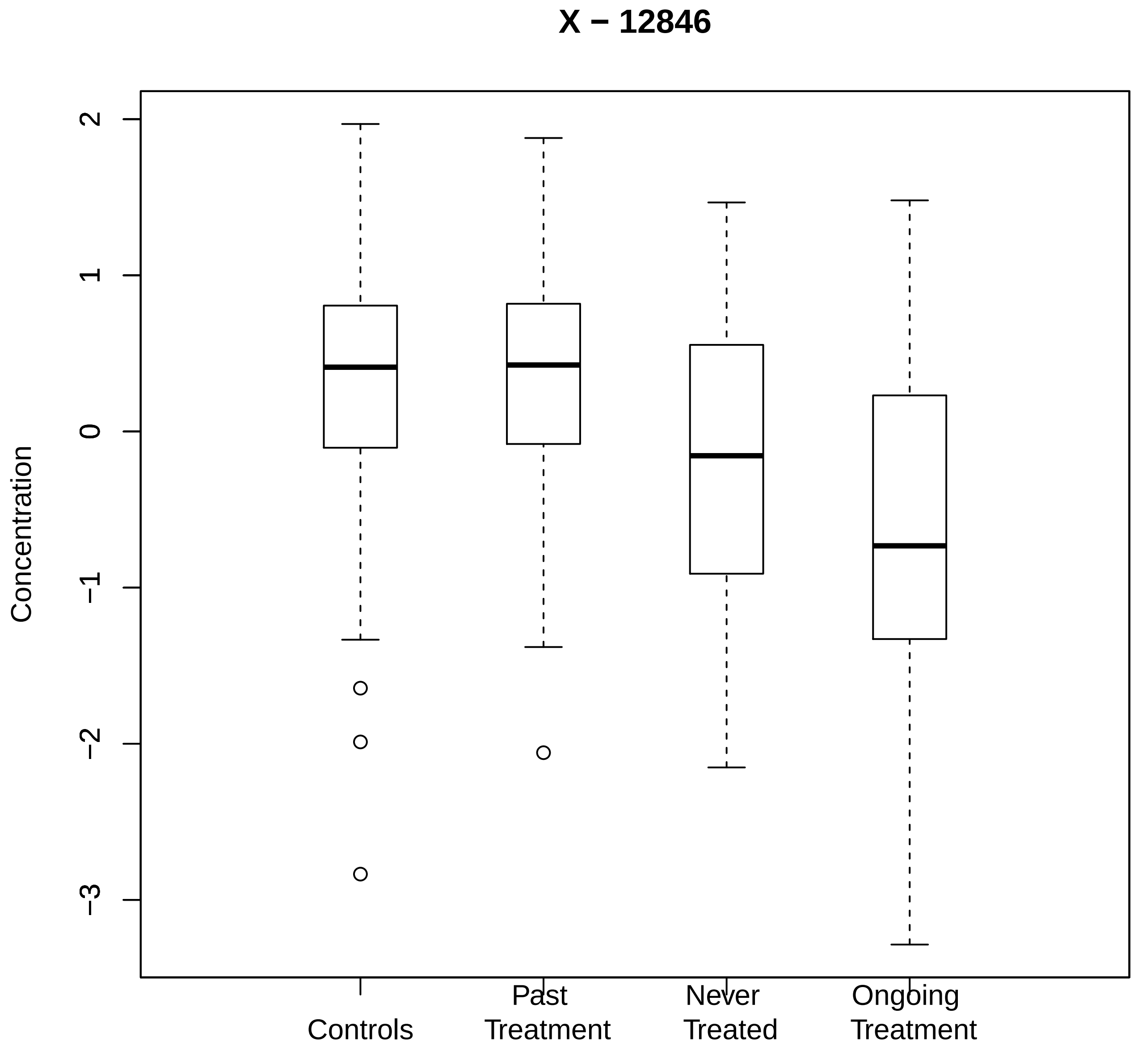

X − 12846
Concentration
2
1
0
−1
−2
−3
Controls
Past Treatment
Never Treated
Ongoing Treatment

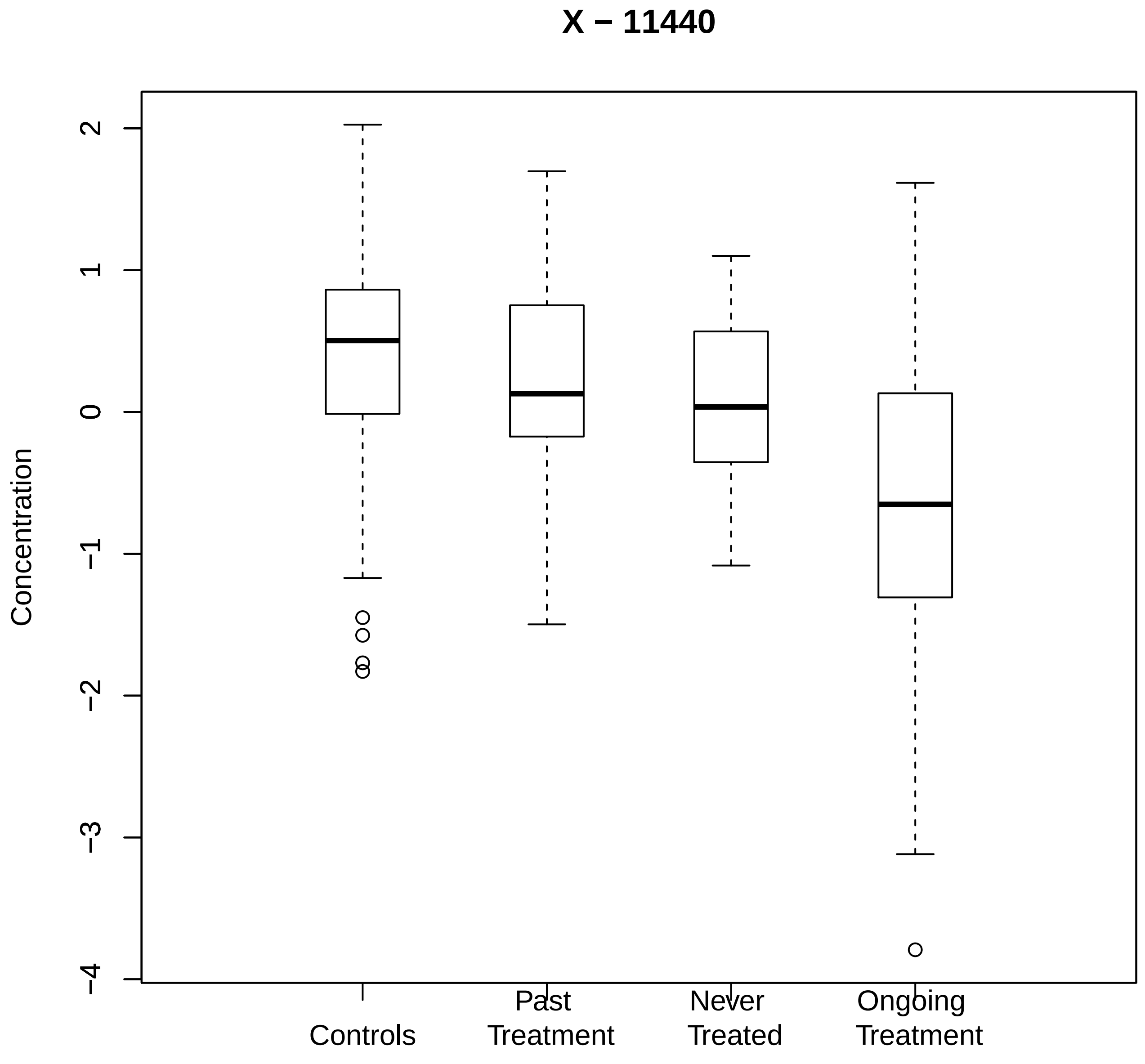
X − 11440
Concentration
2
1
0
−1
−2
−3
−4
Controls
Past Treatment
Never Treated
Ongoing Treatment

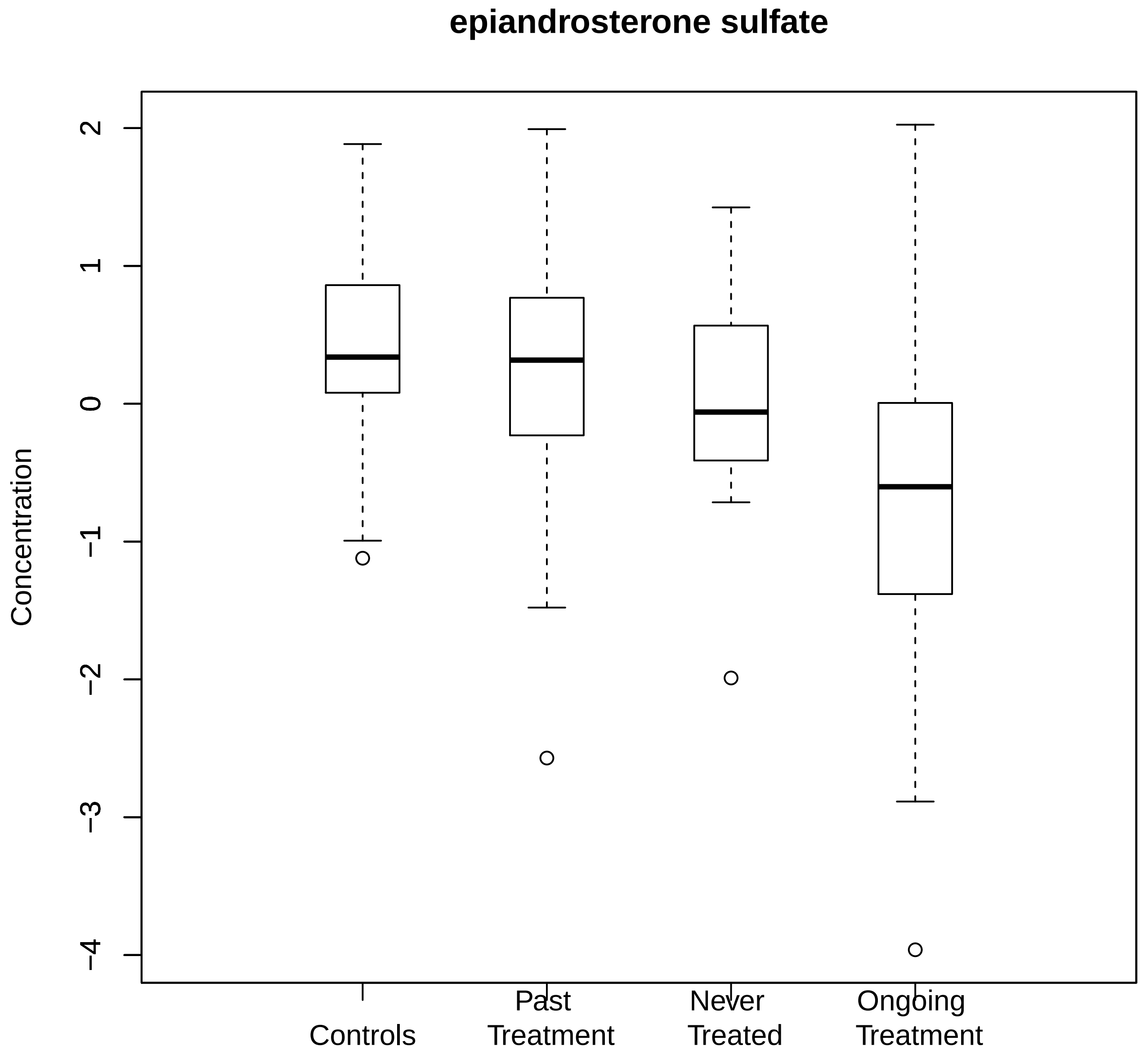
epiandrosterone sulfate
Concentration
2
1
0
−1
−2
−3
−4
Controls
Past Treatment
Never Treated
Ongoing Treatment

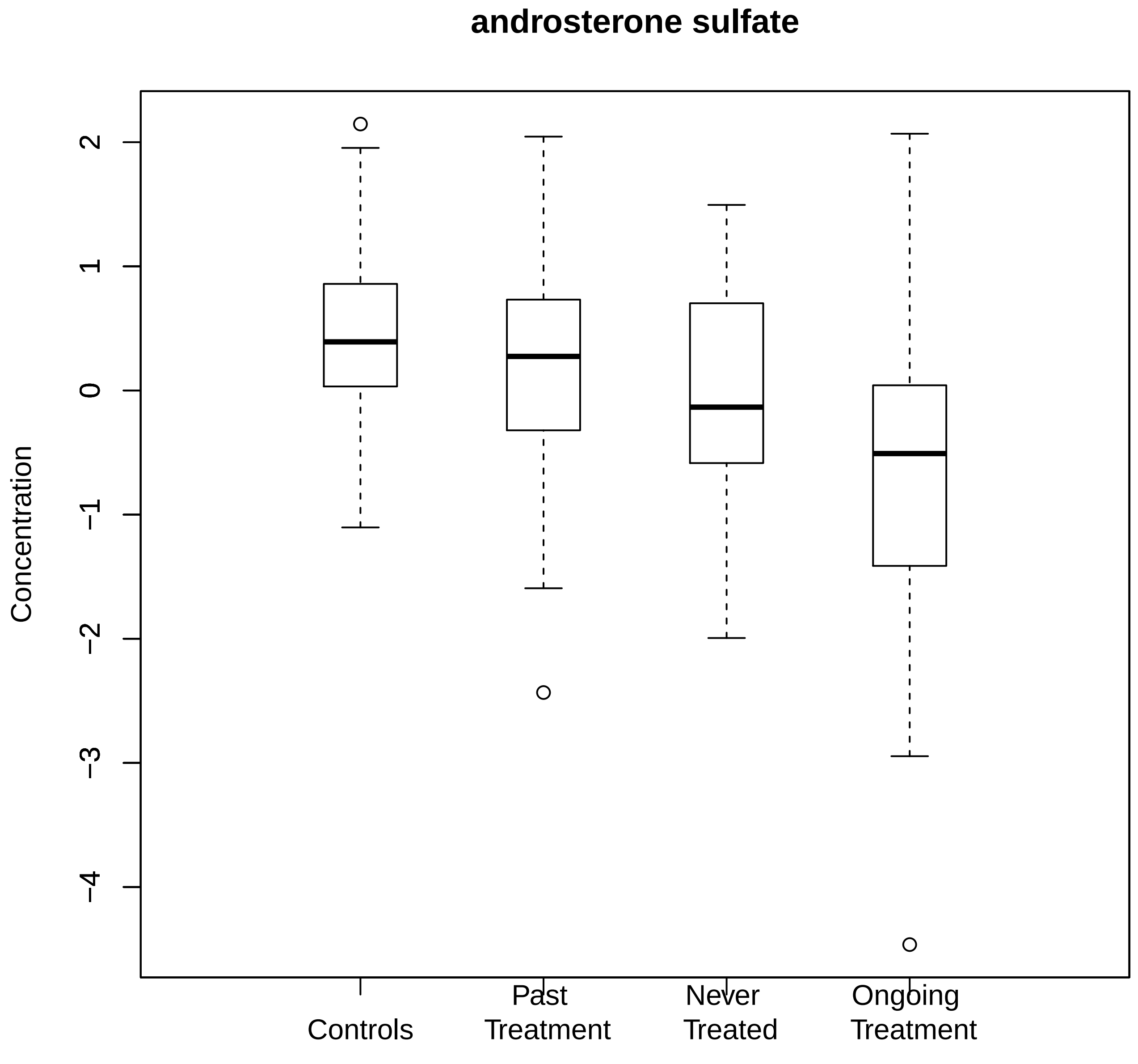
androsterone sulfate
Concentration
2
1
0
−1
−2
−3
−4
Controls
Past Treatment
Never Treated
Ongoing Treatment

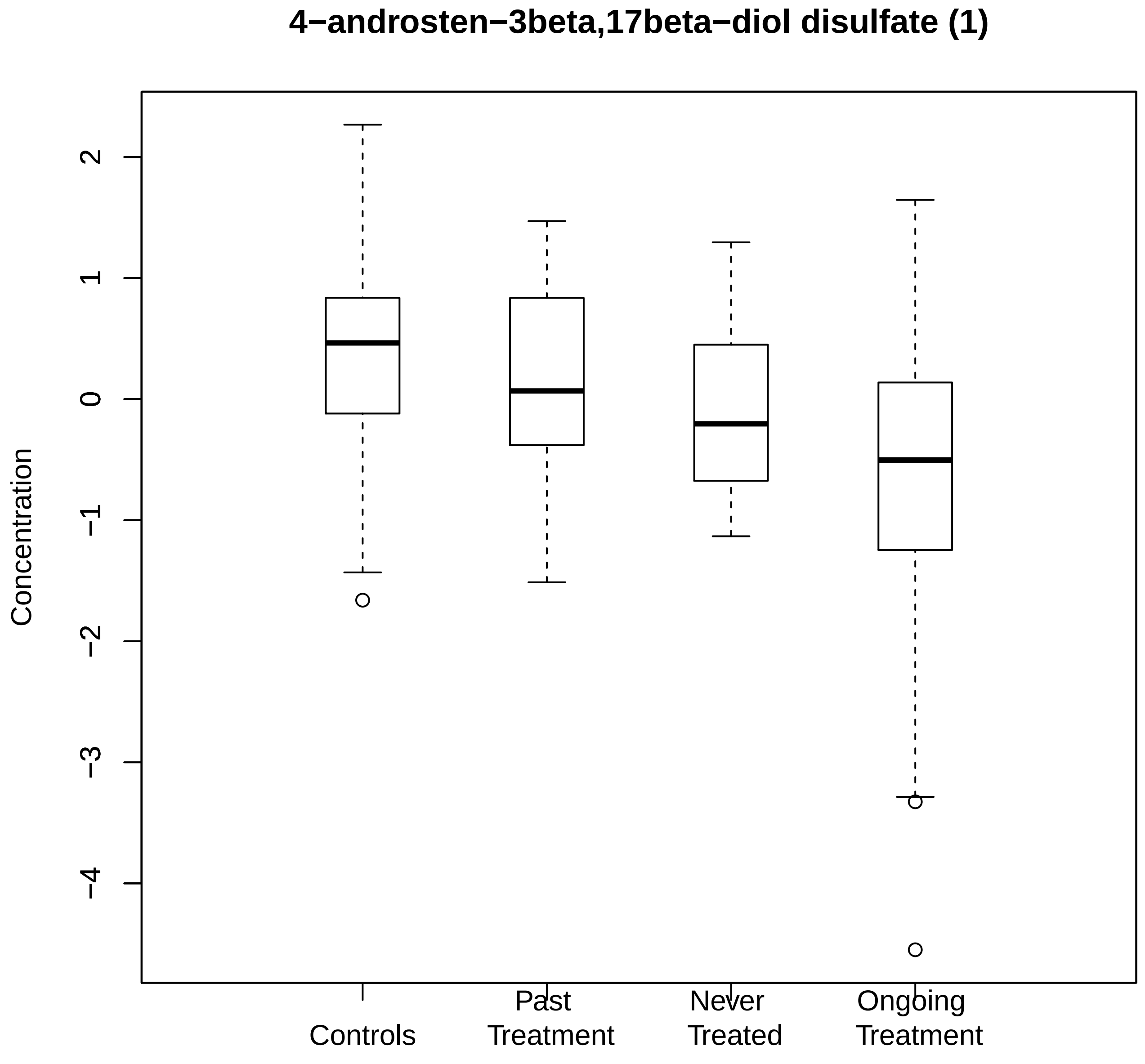

4−androsten−3beta,17beta−diol disulfate (1)
Concentration
2
1
0
−1
−2
−3
−4
Controls
Past Treatment
Never Treated
Ongoing Treatment

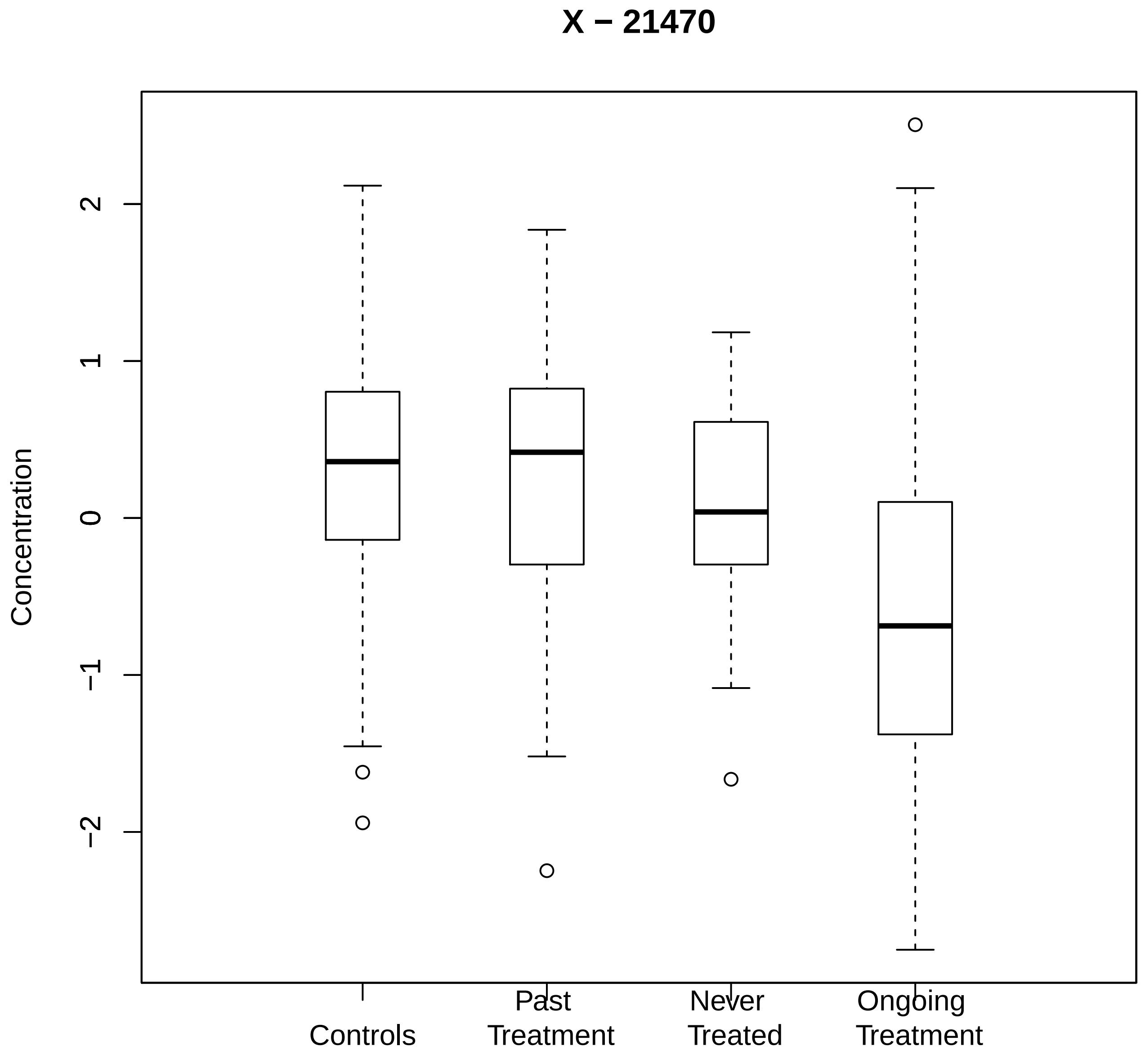
X − 21470
Concentration
2
1
0
−1
−2
Controls
Past
Treatment
Never
Treated
Ongoing
Treatment

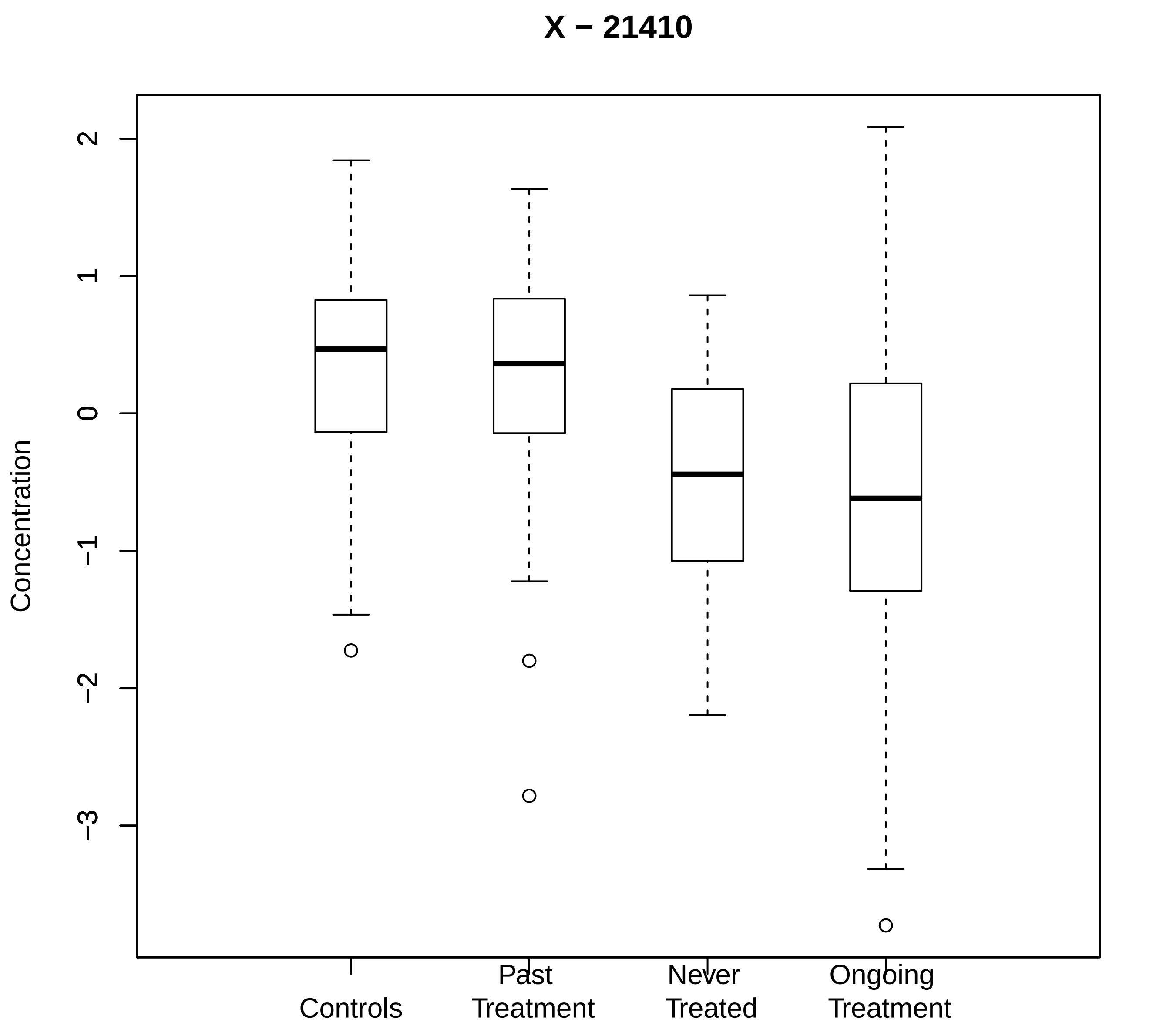

X − 21410
Concentration
2
1
0
−1
−2
−3
Controls
Past Treatment
Never Treated
Ongoing Treatment

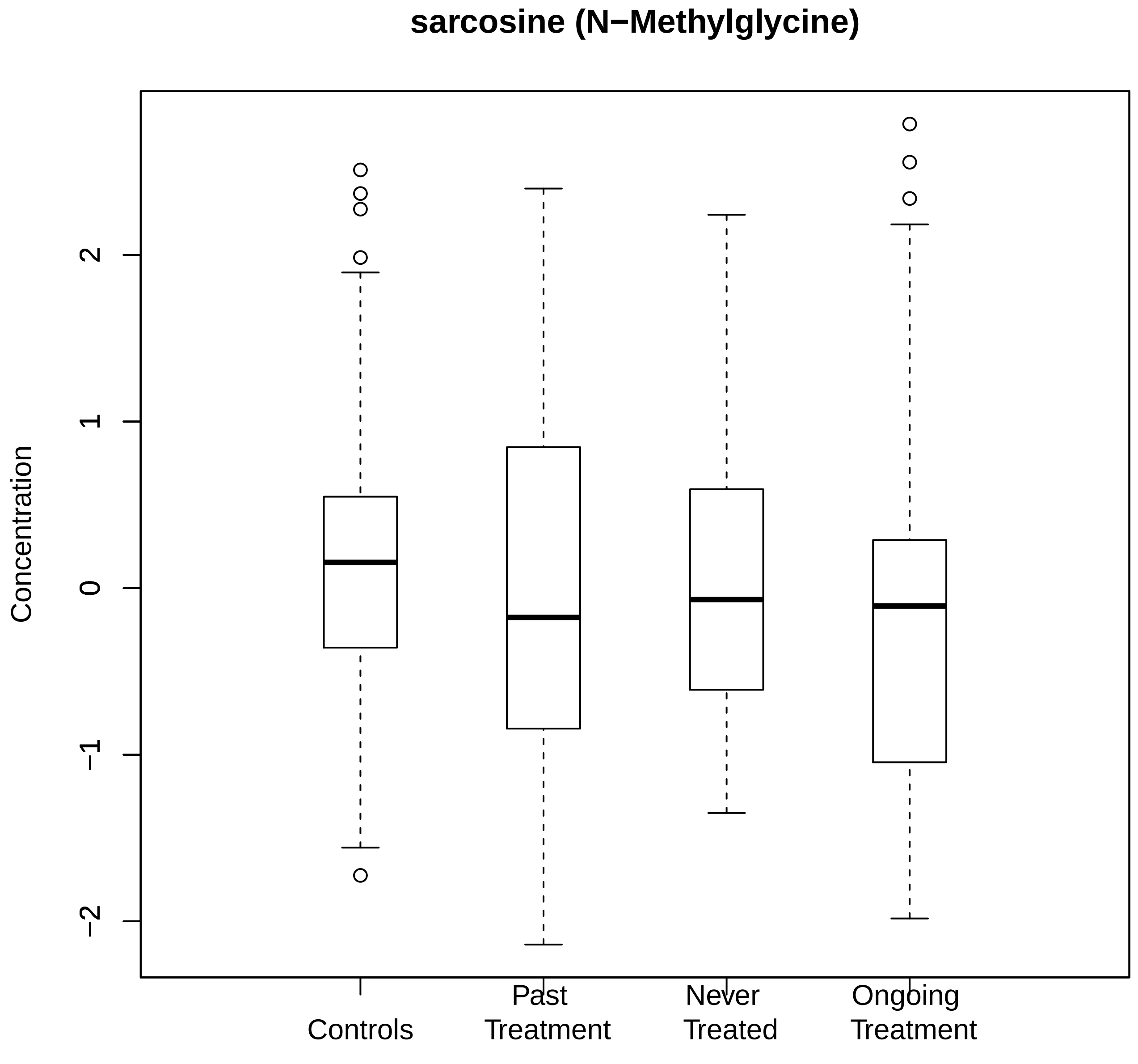
sarcosine (N−Methylglycine)
Concentration
2
1
0
−1
−2
Controls
Past Treatment
Never Treated
Ongoing Treatment

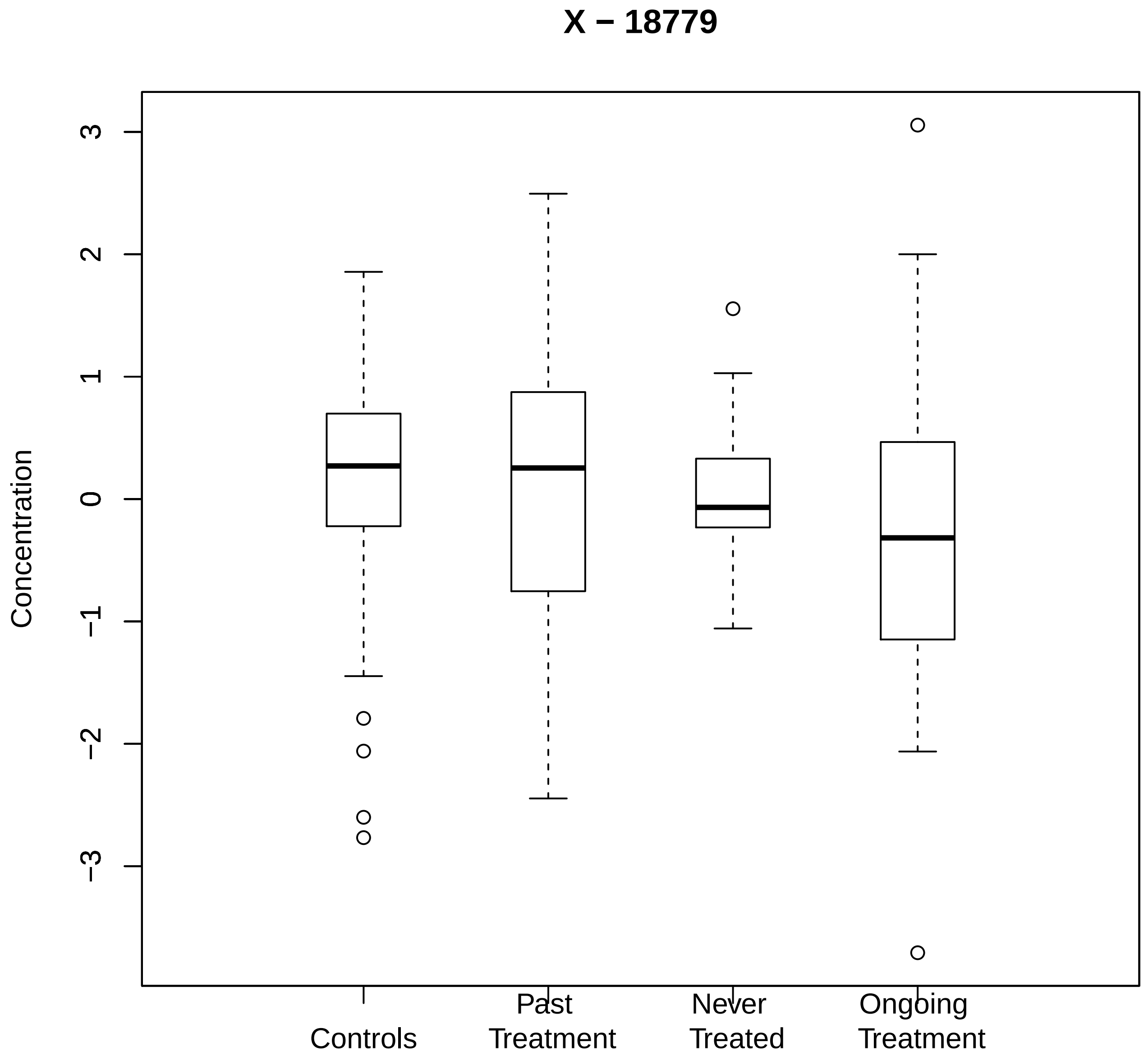
X − 18779
Concentration
3
2
1
0
−1
−2
−3
Controls
Past Treatment
Never Treated
Ongoing Treatment

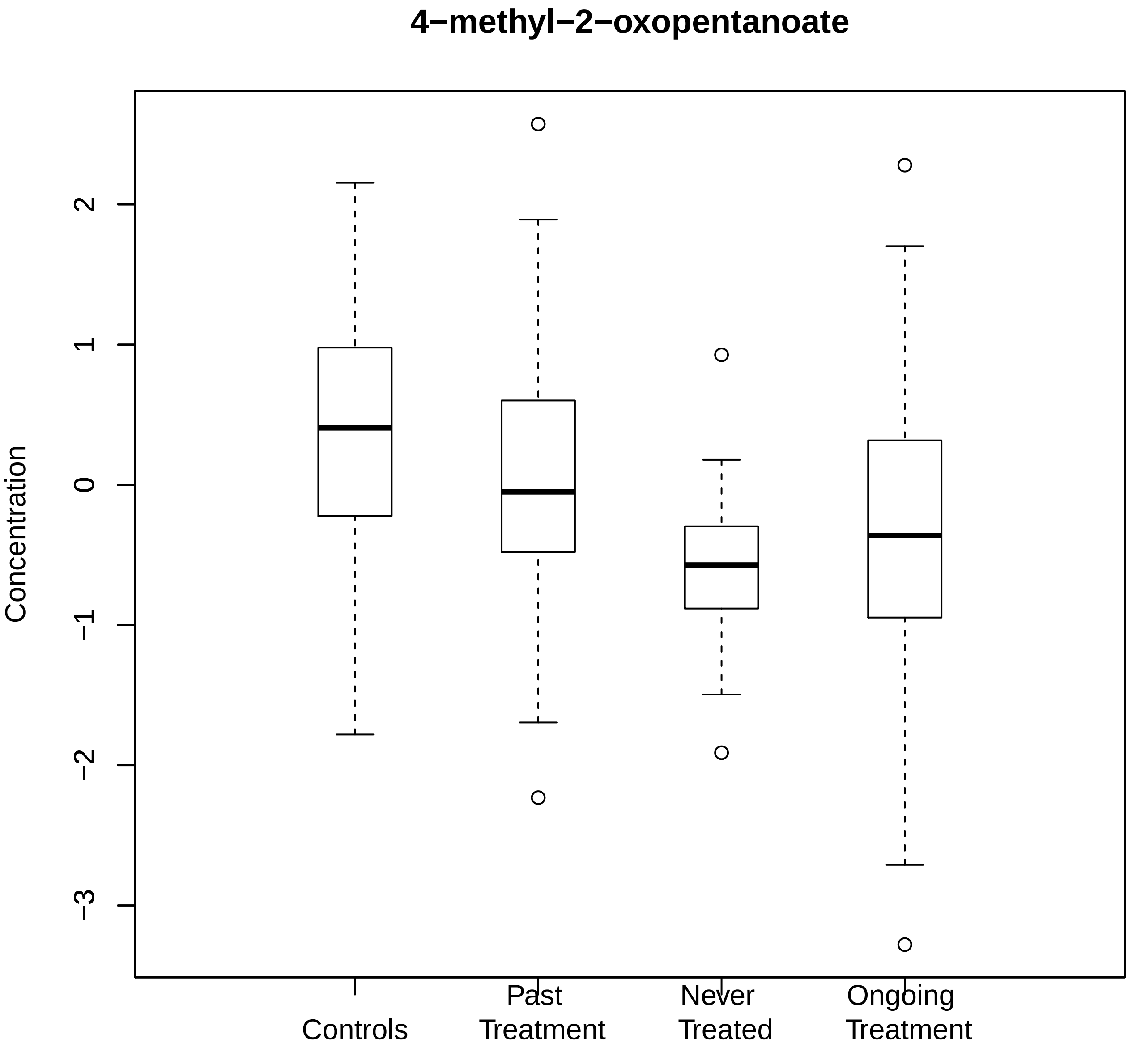
4−methyl−2−oxopentanoate
Concentration
2
1
0
−1
−2
−3
Controls
Past
Treatment
Never
Treated
Ongoing
Treatment

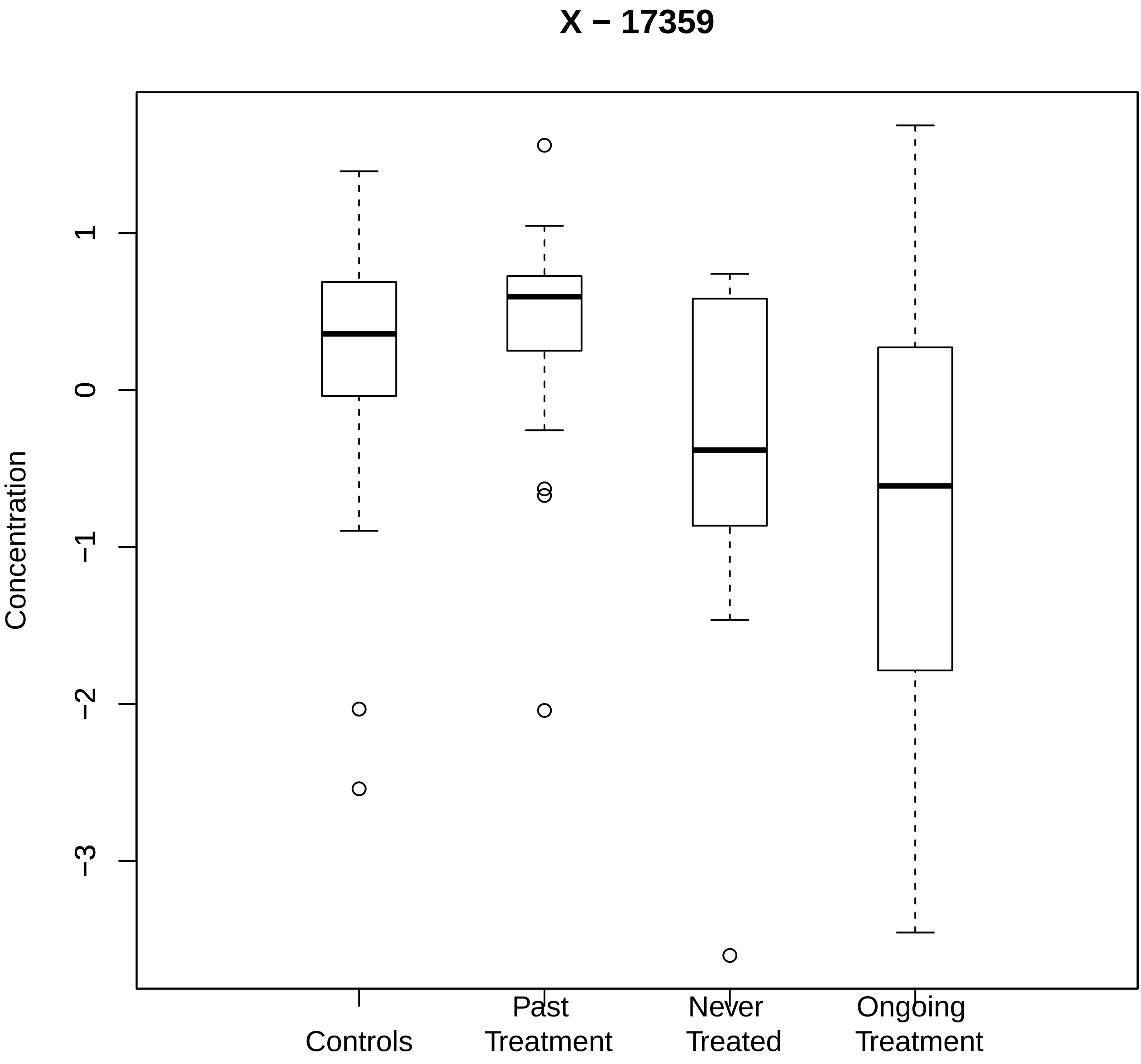
X − 17359
Concentration
1
0
−1
−2
−3
Controls
Past
Treatment
Never
Treated
Ongoing
Treatment

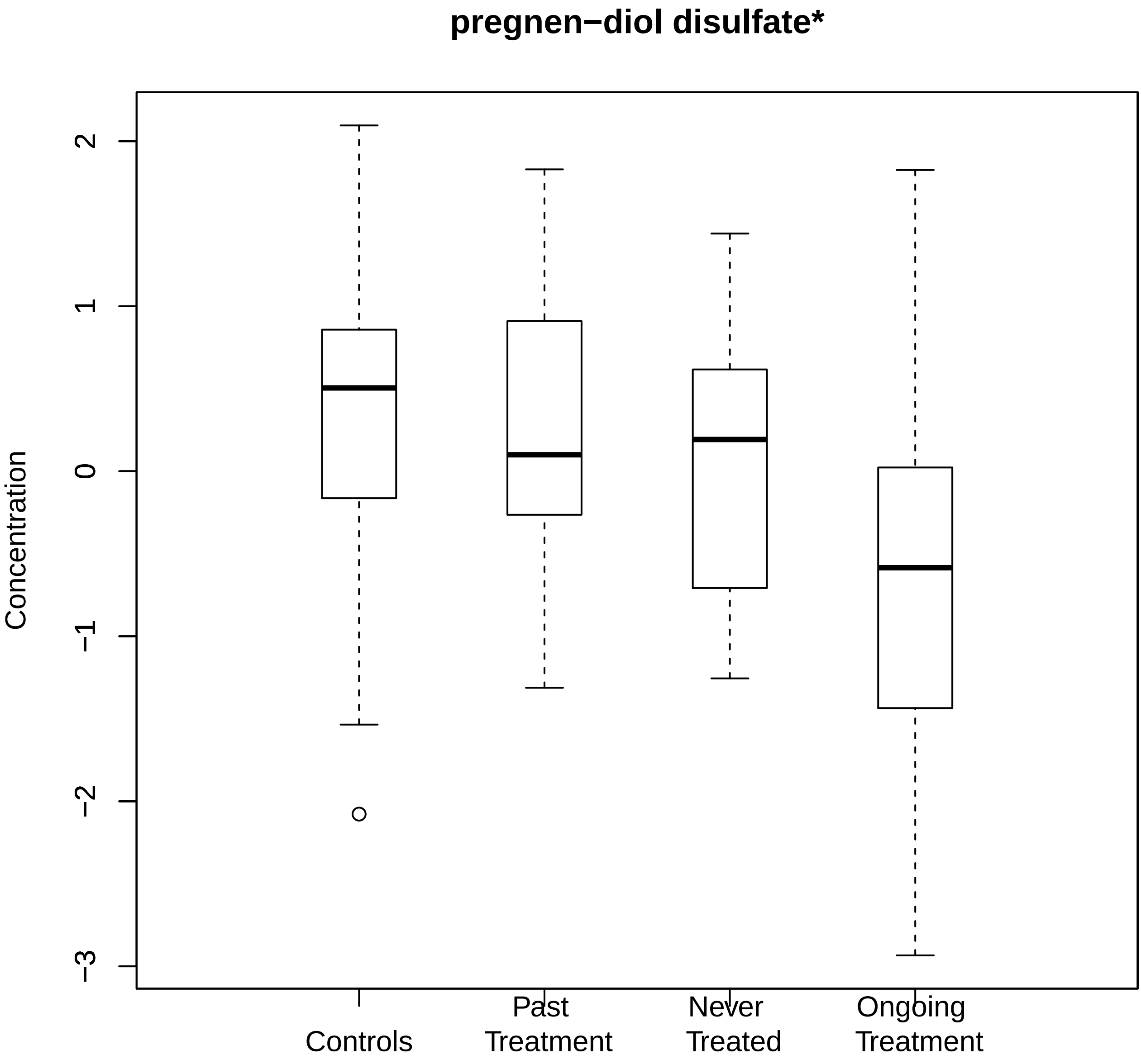
pregnen−diol disulfate*
Concentration
2
1
0
−1
−2
−3
Controls
Past Treatment
Never Treated
Ongoing Treatment

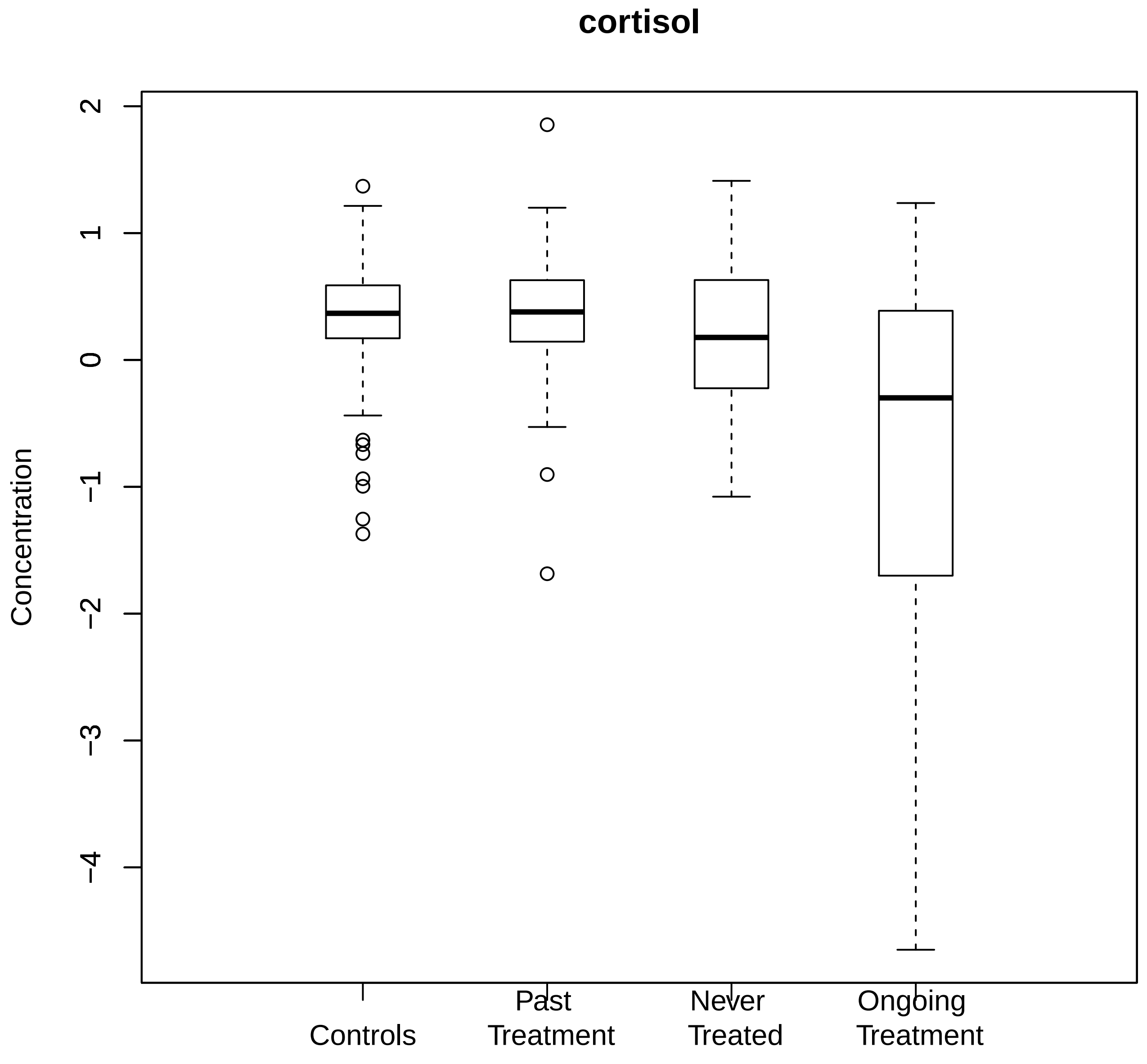
cortisol
Concentration
2
1
0
−1
−2
−3
−4
Controls
Past Treatment
Never Treated
Ongoing Treatment

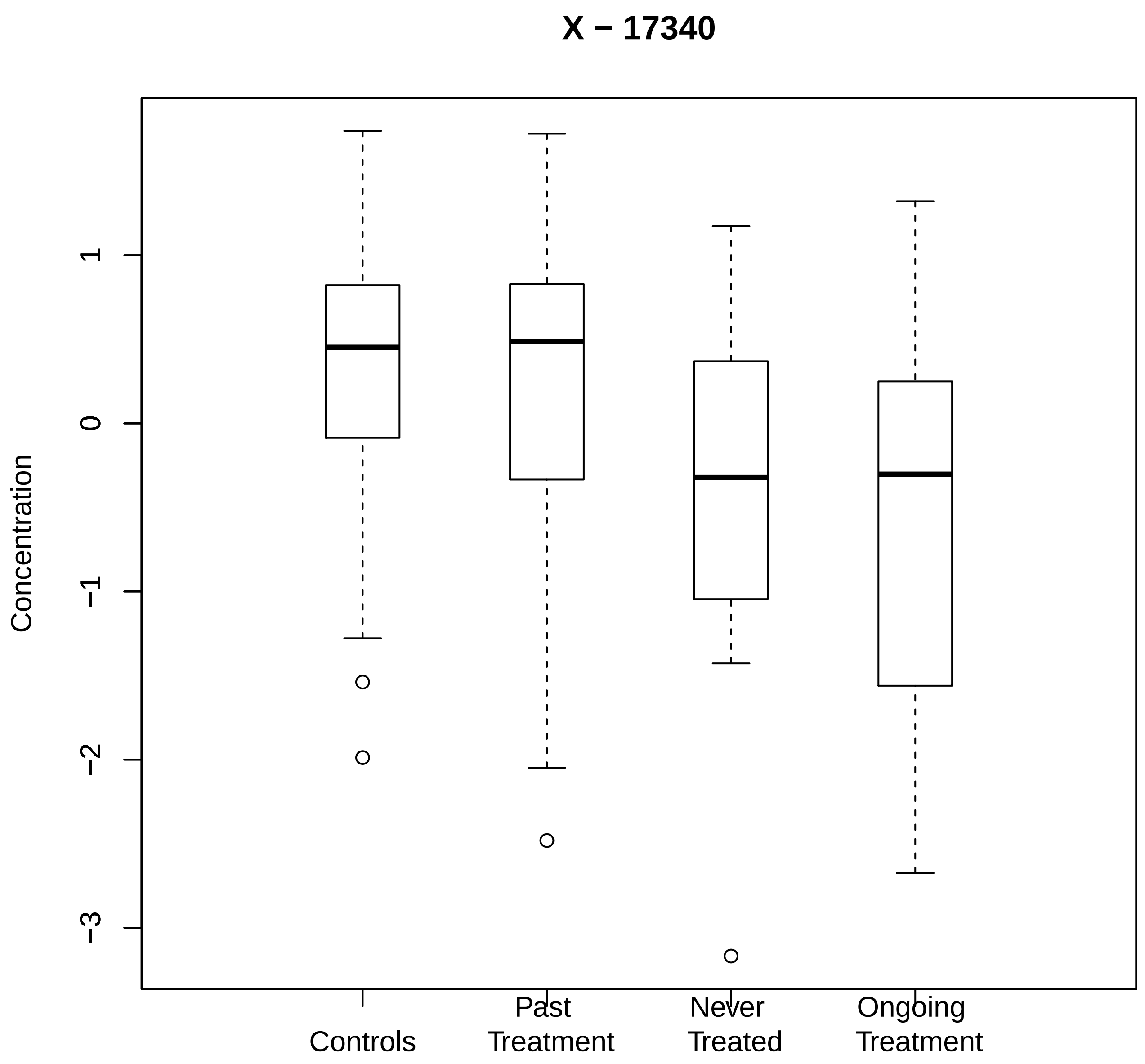
X − 17340
Concentration
1
0
−1
−2
−3
Controls
Past
Treatment
Never
Treated
Ongoing
Treatment

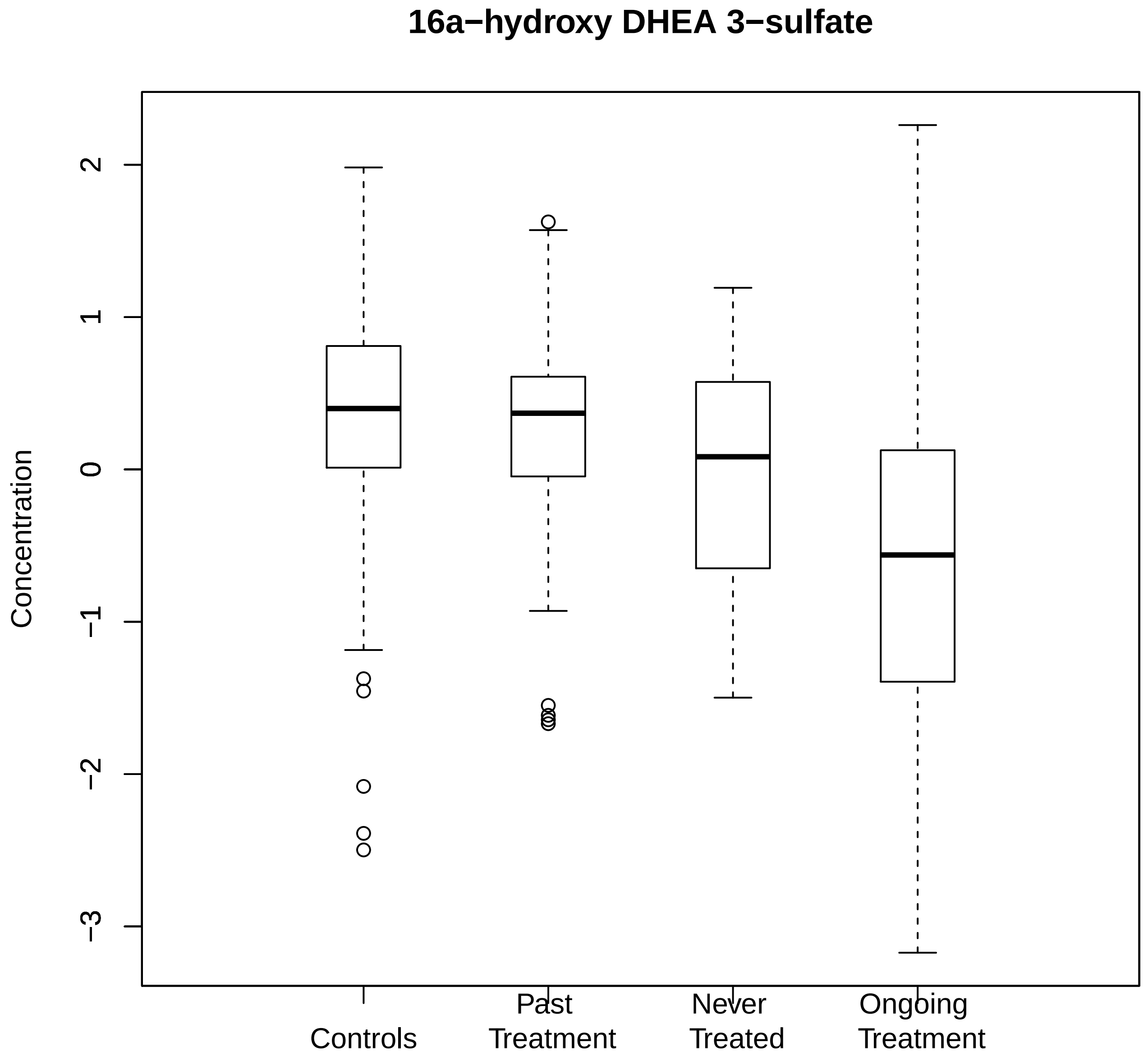

16a−hydroxy DHEA 3−sulfate
Concentration
2
1
0
−1
−2
−3
Controls
Past Treatment
Never Treated
Ongoing Treatment

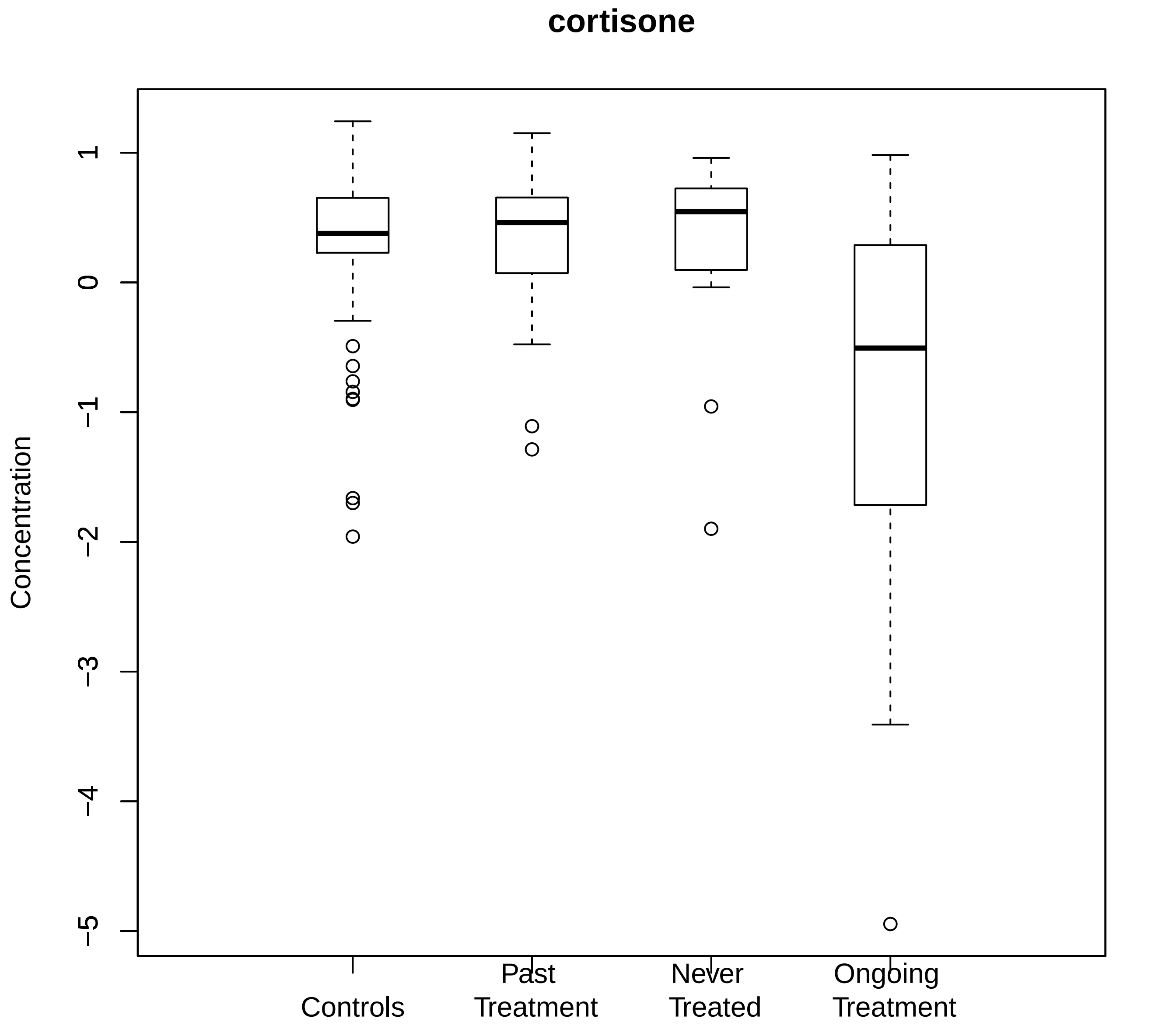

cortisone
Concentration
1
0
−1
−2
−3
−4
−5
Controls
Past
Treatment
Never
Treated
Ongoing
Treatment

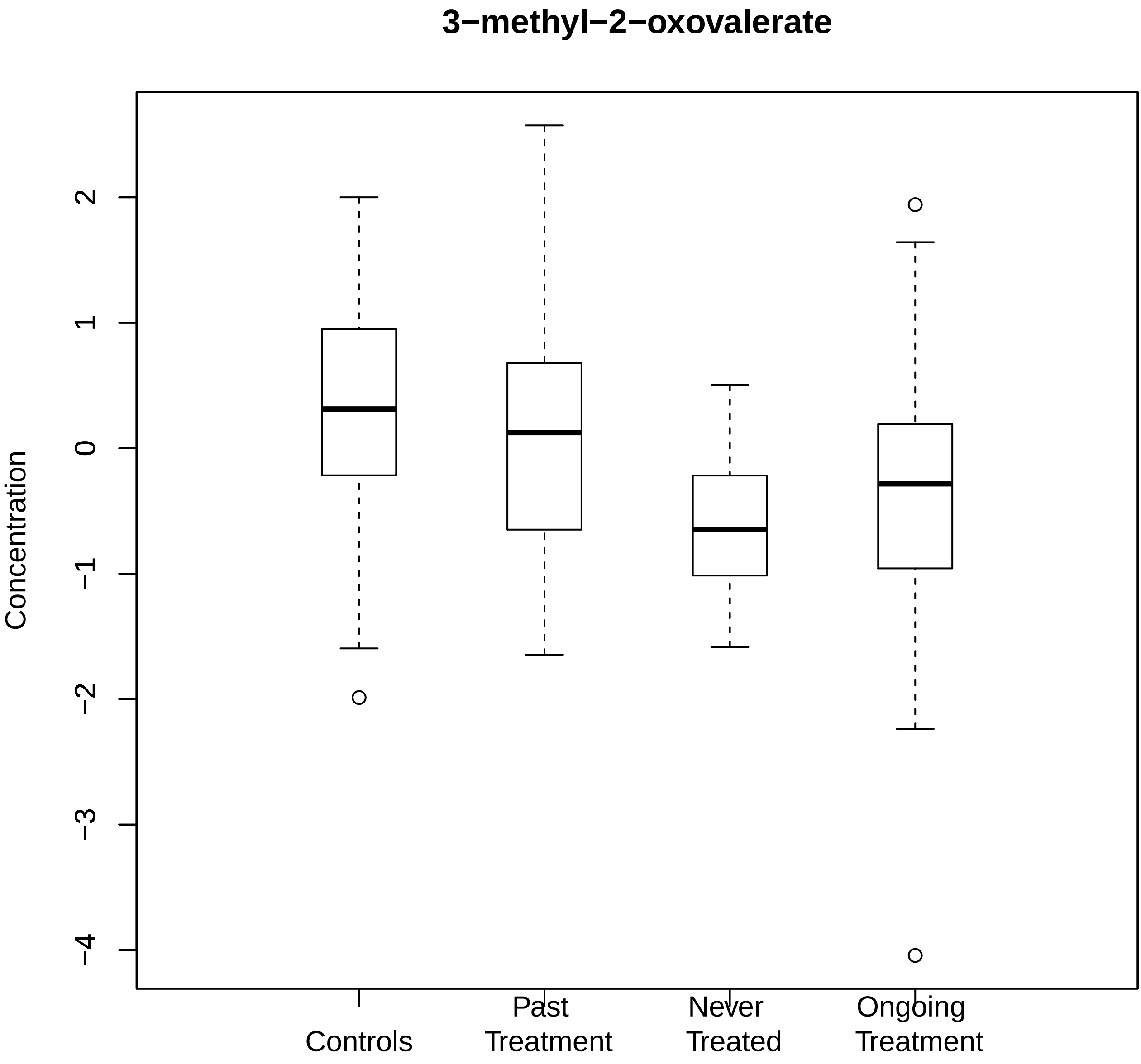
3−methyl−2−oxovalerate
Concentration
2
1
0
−1
−2
−3
−4
Controls
Past Treatment
Never Treated
Ongoing Treatment

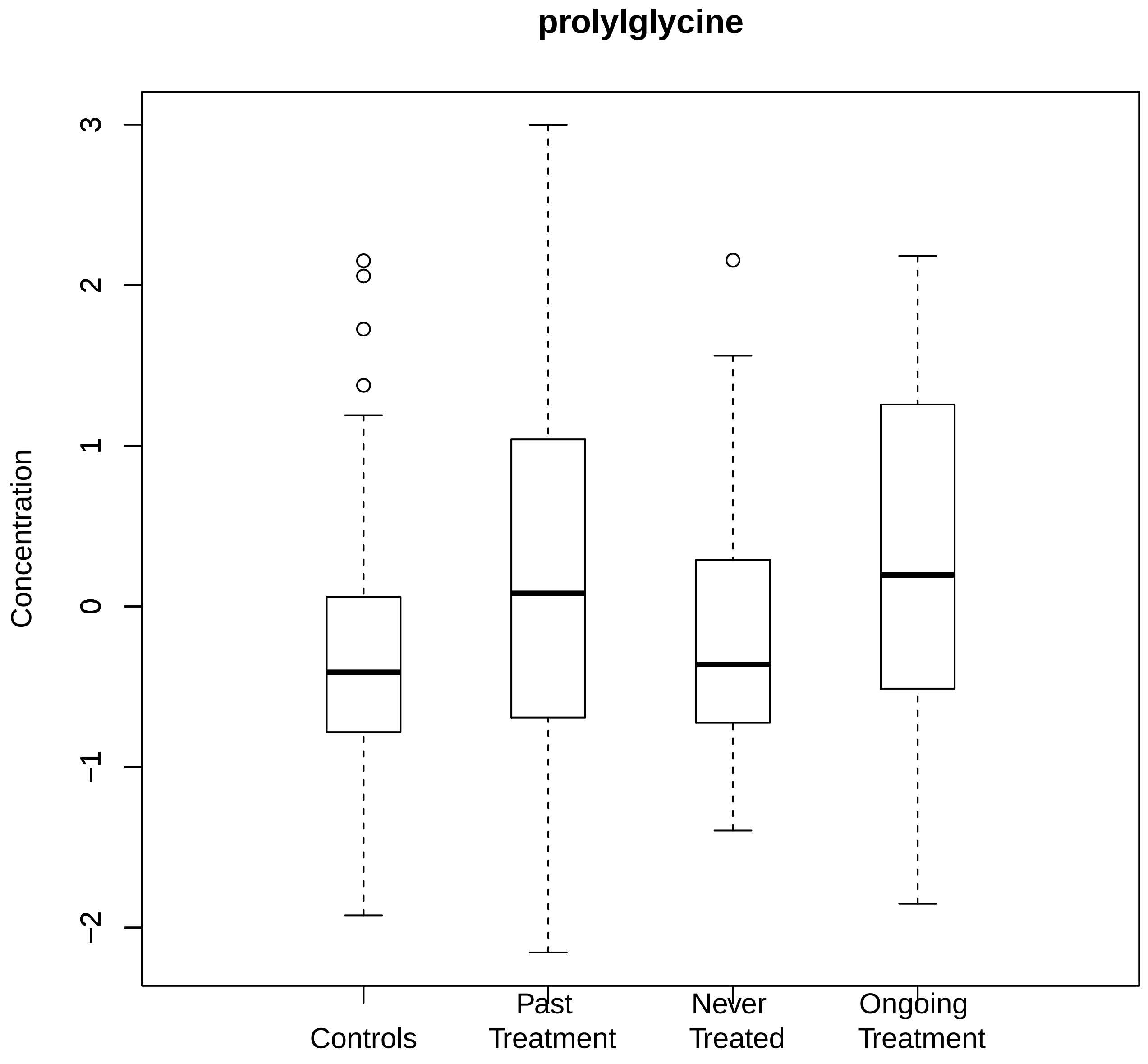

prolylglycine
Concentration
3
2
1
0
−1
−2
Controls
Past Treatment
Never Treated
Ongoing Treatment

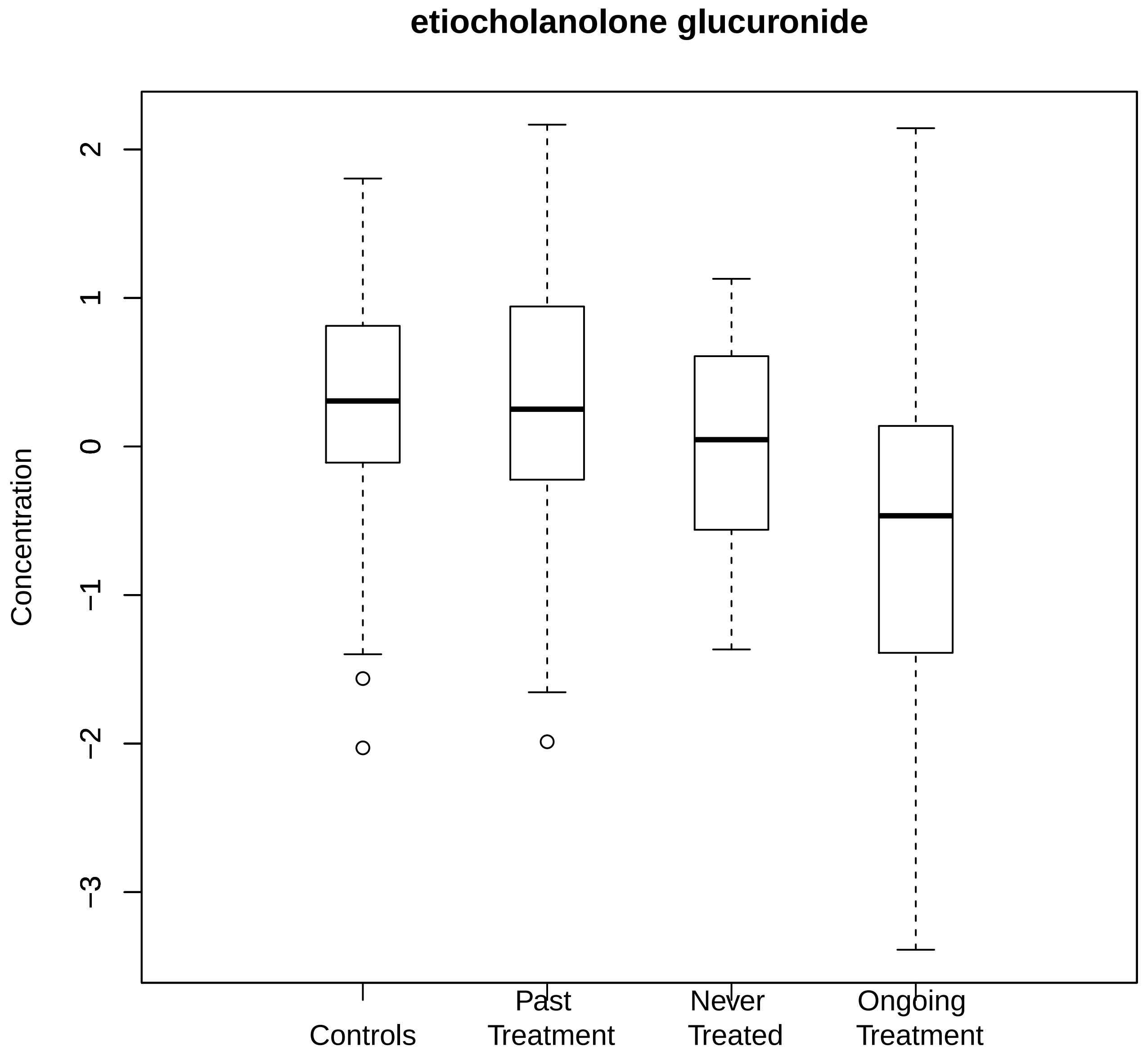

etiocholanolone glucuronide
Concentration
2
1
0
−1
−2
−3
Controls
Past Treatment
Never Treated
Ongoing Treatment

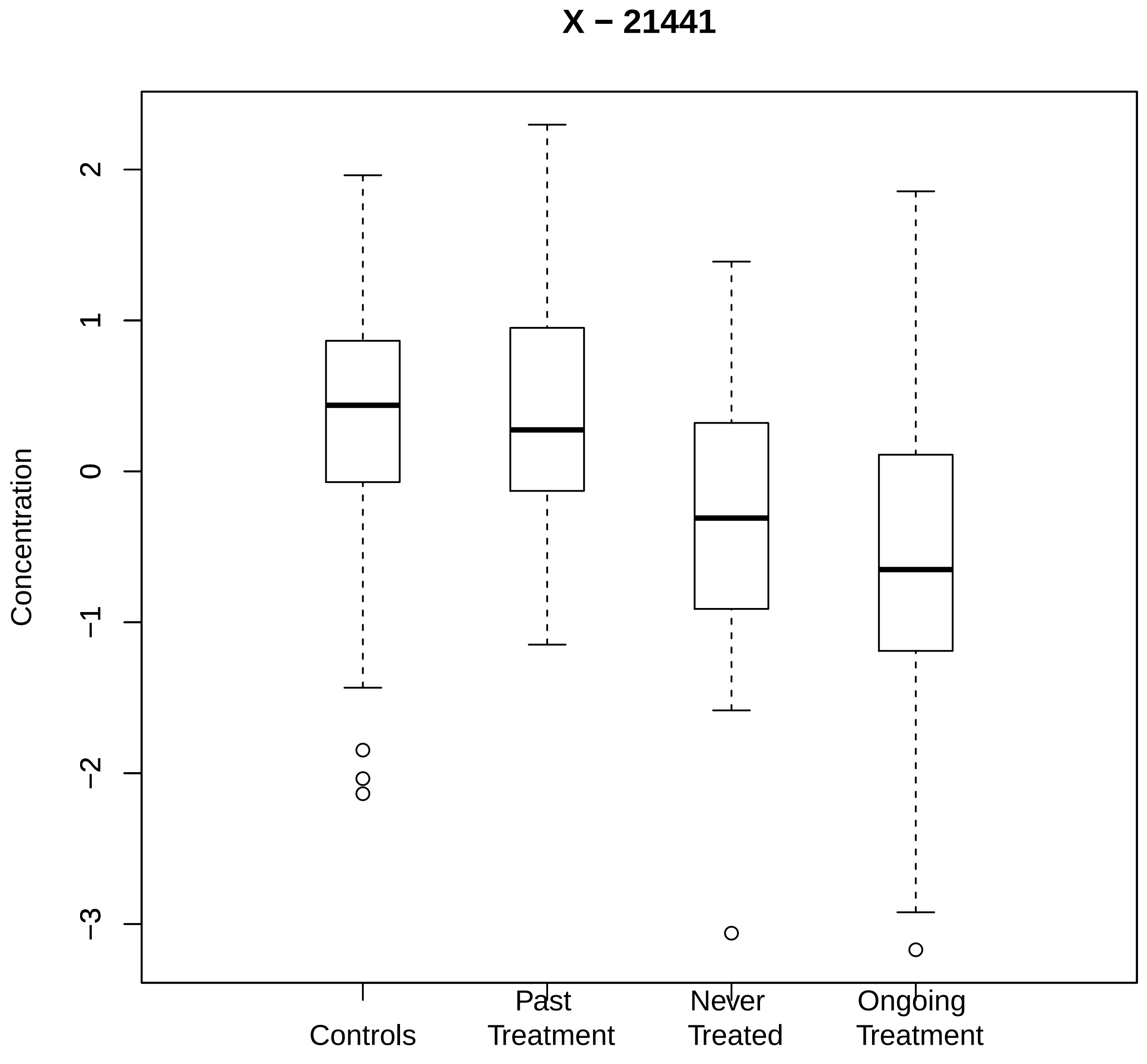
X − 21441
Concentration
2
1
0
−1
−2
−3
Controls
Past Treatment
Never Treated
Ongoing Treatment

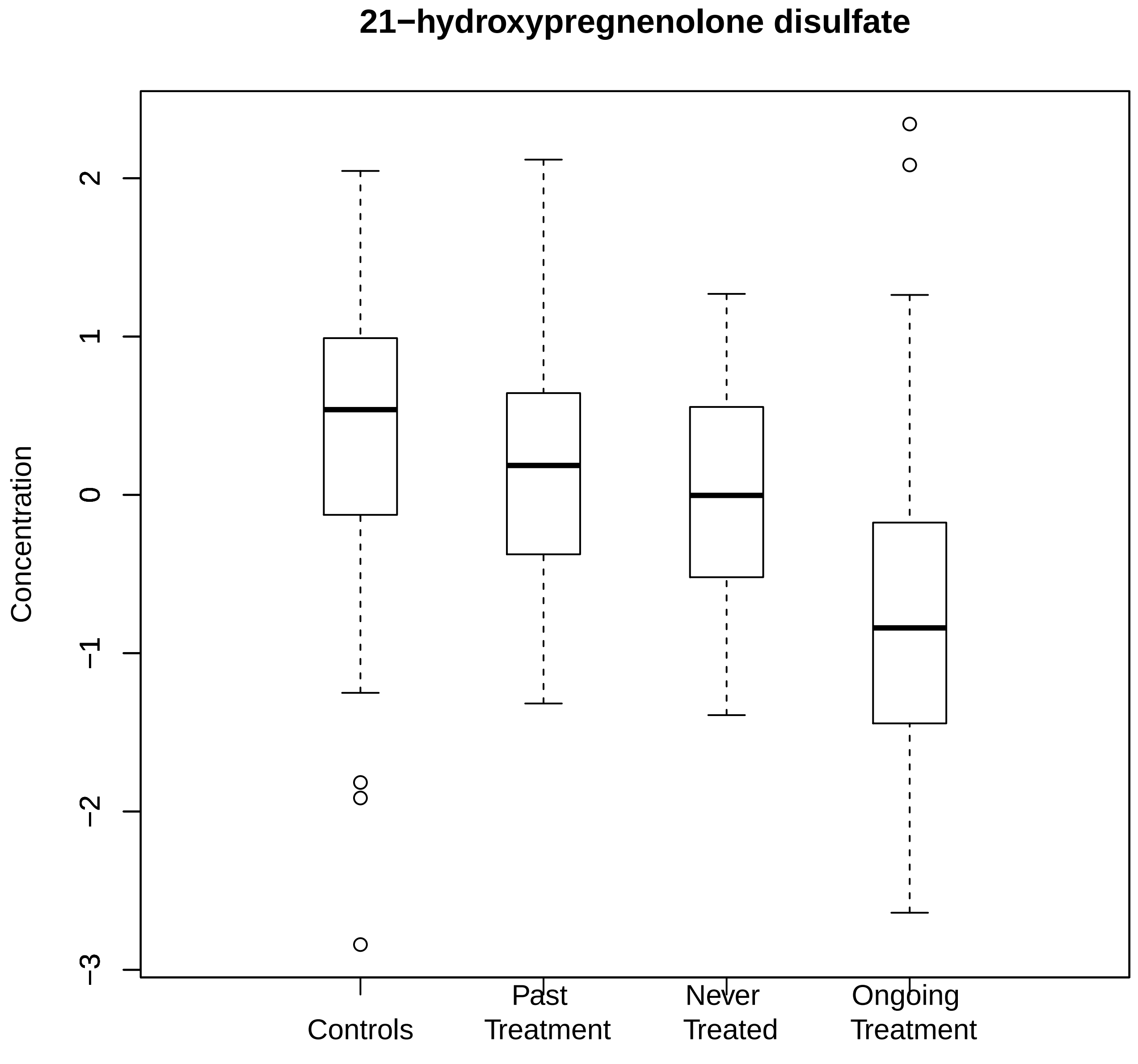
21−hydroxypregnenolone disulfate
Concentration
2
1
0
−1
−2
−3
Controls
Past Treatment
Never Treated
Ongoing Treatment

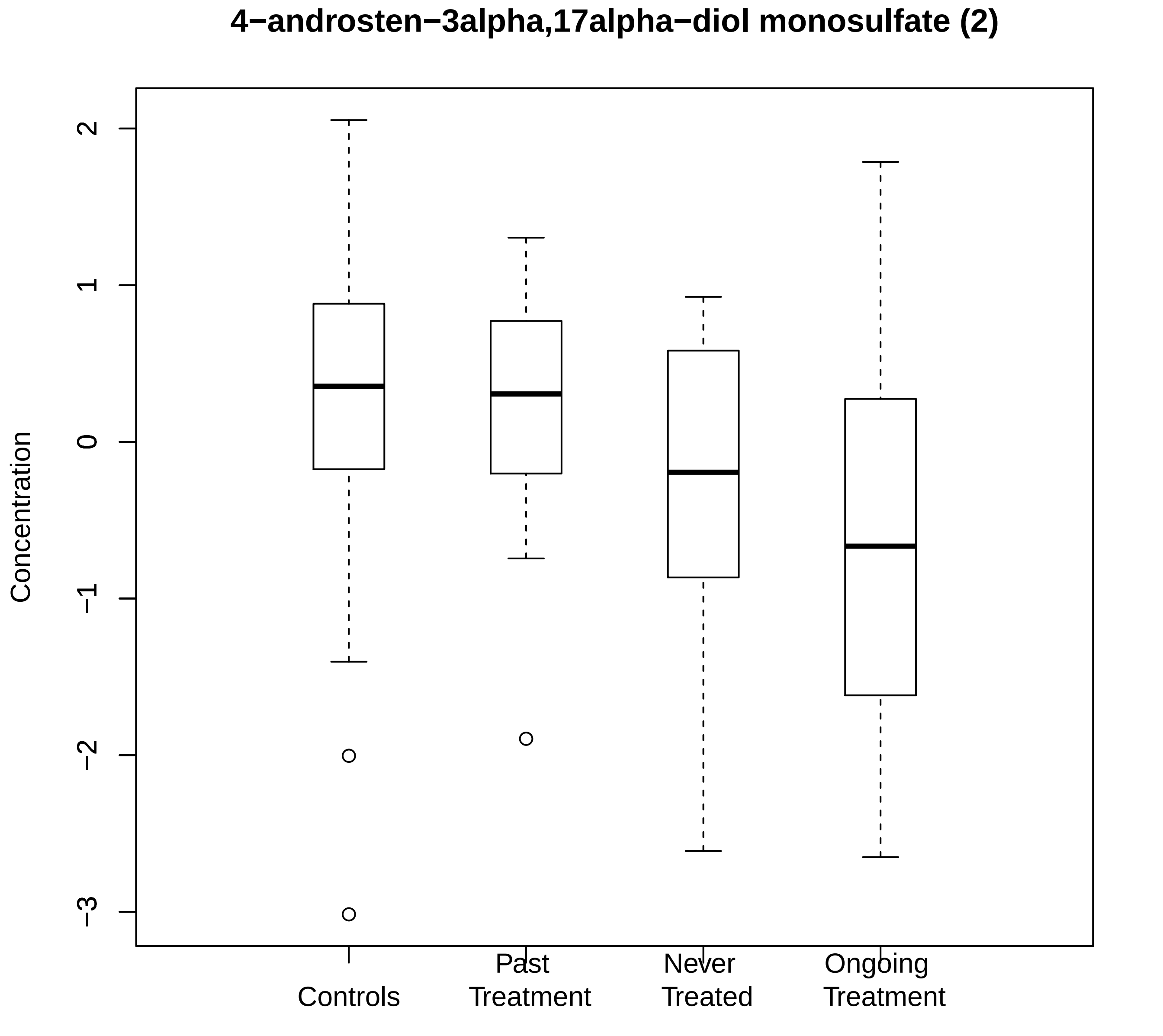

4−androsten−3alpha,17alpha−diol monosulfate (2)
Concentration
2
1
0
−1
−2
−3
Controls
Past Treatment
Never Treated
Ongoing Treatment

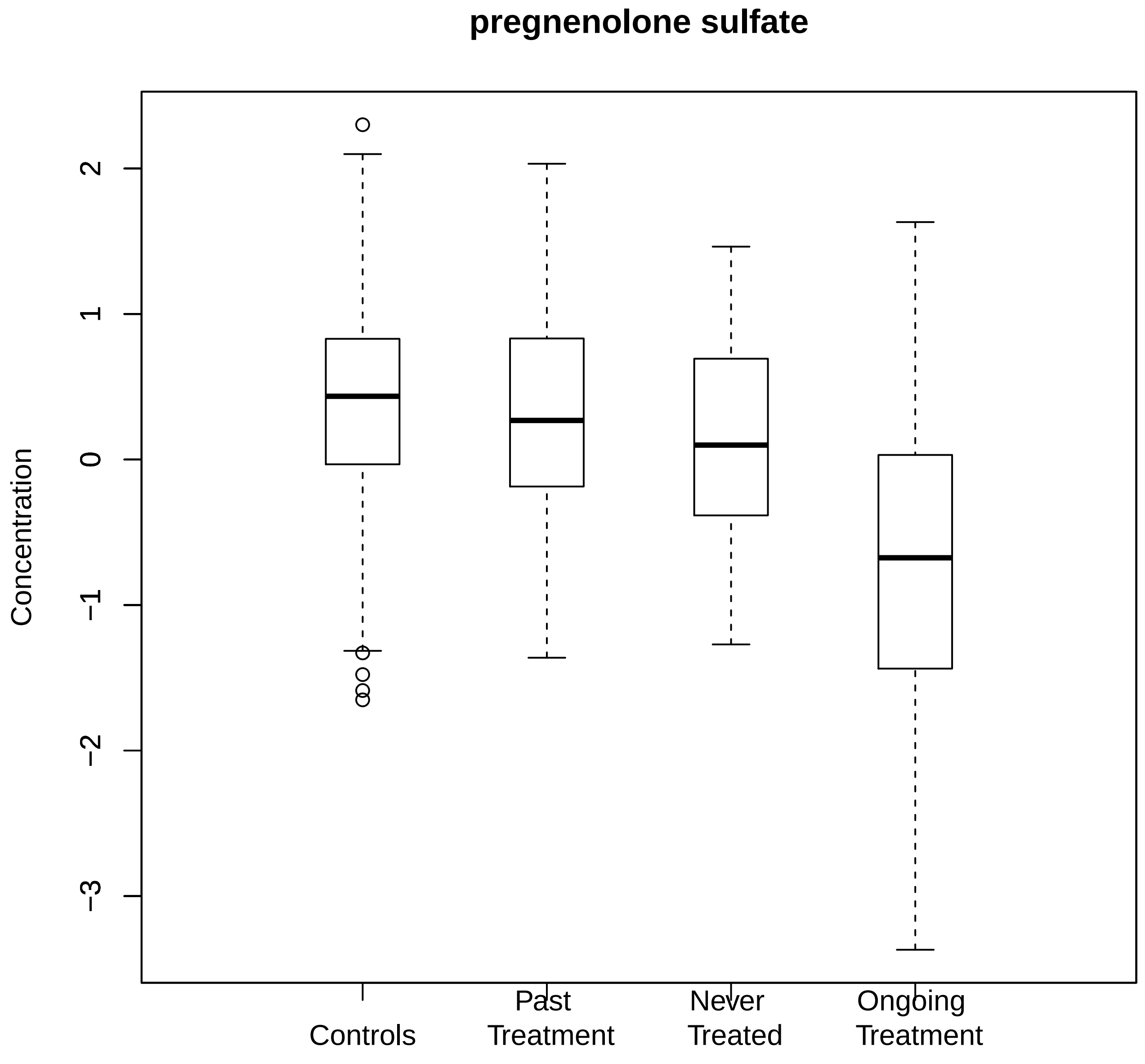
pregnenolone sulfate
Concentration
2
1
0
−1
−2
−3
Controls
Past Treatment
Never Treated
Ongoing Treatment
